# Xenon binding by a tight yet adaptive chiral soft capsule

**DOI:** 10.1038/s41467-020-20081-8

**Published:** 2020-12-07

**Authors:** Shi-Xin Nie, Hao Guo, Teng-Yu Huang, Yu-Fei Ao, De-Xian Wang, Qi-Qiang Wang

**Affiliations:** 1 Beijing National Laboratory for Molecular Sciences, CAS Key Laboratory of Molecular Recognition and Function, Institute of Chemistry, Chinese Academy of Sciences, 100190 Beijing, China; 2 University of Chinese Academy of Sciences, 100049 Beijing, China

**Keywords:** Organic chemistry, Molecular capsules

## Abstract

Xenon binding has attracted interest due to the potential for xenon separation and emerging applications in magnetic resonance imaging. Compared to their covalent counterparts, assembled hosts that are able to effectively bind xenon are rare. Here, we report a tight yet soft chiral macrocycle dimeric capsule for efficient and adaptive xenon binding in both crystal form and solution. The chiral bisurea-bisthiourea macrocycle can be easily synthesized in multi-gram scale. Through assembly, the flexible macrocycles are locked in a bowl-shaped conformation and buckled to each other, wrapping up a tight, completely sealed yet adjustable cavity suitable for xenon, with a very high affinity for an assembled host. A slow-exchange process and drastic spectral changes are observed in both ^1^H and ^129^Xe NMR. With the easy synthesis, modification and reversible characteristics, we believe the robust yet adaptive assembly system may find applications in xenon sequestration and magnetic resonance imaging-based biosensing.

## Abstract (web summary)

Xenon binding carries potential for xenon separation and emerging applications in magnetic resonance imaging. Here, the authors report a rare example of a tight yet soft capsule, assembled from two chiral bisurea-bisthiourea macrocycle components, that can efficiently and adaptively bind xenon in both the solid state and solution.

## Introduction

Xenon is a hydrophobic, highly polarizable but rather inert gas. The ^129^Xe isotope (natural abundance of 26.4%) has a spin of *I* = 1/2 and its NMR parameters including chemical shift and exchange kinetics are very sensitive to surrounding environments. Meanwhile, application of hyperpolarization (hp) technique can increase the signal by more than 10^4^-fold, thus allowing for very sensitive detection^1,2^. Consequently, xenon has long been used as an inert probe to determine cavity environments of porous materials and organisms such as proteins^3–6^. Promise has also been shown for xenon-based magnetic resonance imaging (MRI)^7^ and target-specific biosensing when a suitable xenon-trapping host is applied^8–13^.

Even with the important applications, especially recent exciting biosensing applications, our understanding of the host-guest chemistry of xenon is limited and development of efficient xenon receptors is highly desirable. As an inert spherical monoatomic gas, xenon lacks an apparent binding site; van der Waals interaction, specifically London dispersion force, is thought to be the main driving force. This makes xenon binding very challenging. Several macrocycle and cage compounds, including hemicarcerands^14,15^, cyclodextrins^16,17^, calixarenes^18–21^, cryptophanes^22–30^, cucurbiturils^31–37^, and recent porous imine cages^38–40^ have been explored for xenon binding, in solution or solid state. Cryptophanes are shown to be outstanding in terms of high affinity and competence for biosensing applications^8,9,13,41,42^, though, tedious synthesis and sometimes chiral resolution of the intrinsic enantiomers are required for chirality-sensitive detection^43–45^. On the other hand, assembled hosts have the advantages of reversible formation and easy modular accessibility, however, they are barely explored for xenon binding. In the only few reported examples, earlier Rebek’s rigid, hydrogen-bonded “tennis ball” was found to include xenon on ^1^H NMR observation^46^. Recently a cyclic peptide-based columnar assembly was shown able to confine xenon in crystalline form^47^. Meanwhile, metal-assembled Fe_4_L_6_^48^ and Co_4_L_6_^49^ cages were recently shown able to encapsulate xenon, with a binding constant of 16 M^−1^ determined for the former.

Herein we report a stable dimeric assembly of a chiral bisurea-bisthiourea macrocycle and its favorable, adaptive-binding ability toward xenon. The assembly represents a tight, completely sealed yet rather soft capsule and encapsulates xenon with a very high affinity for an assembled host. The adaptive-binding dynamics due to induced fit of the surrounding macrocyclic skeletons is revealed not only in crystal form, but also in solution through the unique spectral features of the dimeric assembly.

## Results

### Design and synthesis

**Fig. 1:**
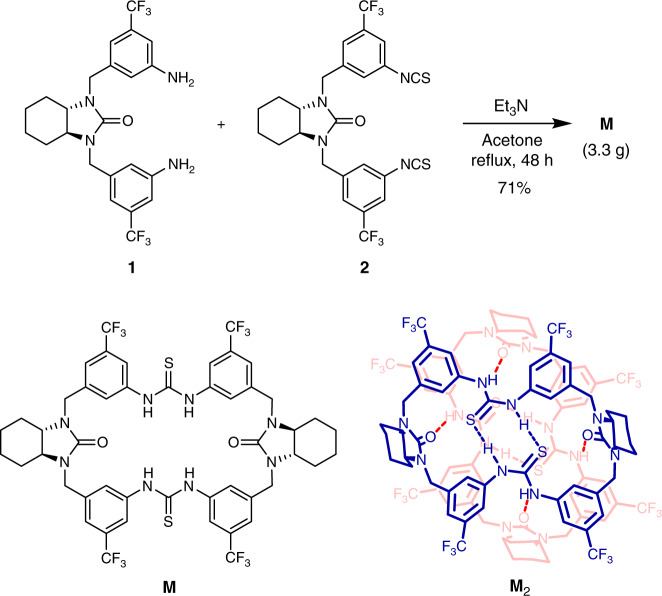
Synthesis of the chiral bisurea-bisthiourea macrocycle M. The structures of the macrocycle and its hydrogen-bonded dimeric form are drawn in the bottom.

Recently we have developed a set of chiral tetraamino-bisthiourea macrocycles for substrate-induced assembly asymmetric catalysis^50^. We envisioned that introducing additional carbonyl groups to the diamine moieties on both sides will lead to a type of bisurea-bisthiourea chiral macrocycle **M** which incorporates multiple hydrogen bond donors (NH) and acceptors (C=S, C=O) simultaneously, and would favor intra- or intermolecular assembly (Fig. 1). The synthesis of the macrocycle is straightforward. After several simple transformations, the 1 + 1 condensation between bis-amine **1** and bis-isothiocyanate **2** furnished multi-grams of the chiral macrocycle in one batch with an excellent yield of 71% (Fig. 1 and Supplementary Methods).

### Structures of the dimeric capsule

**Fig. 2:**
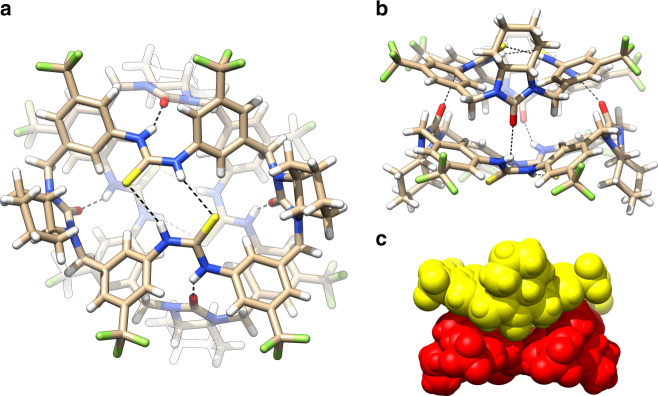
Crystal structure of the dimeric capsule M_2_. **a** Top view. **b** Side view. **c** Space filling view. Only the first form (Form 1) of the two crystallographically independent dimeric capsules is shown. H-bonding distances (given by the heavy atom distances): C=O∙∙∙H–N 2.808, 2.832, 2.808, and 2.832 Å; C=S∙∙∙H–N 3.393, 3.399, 3.393, and 3.399 Å.

High-quality crystals of the dimeric capsule **M**_2_ were reproducibly obtained by diffusion of *n*-hexane to a macrocycle toluene solution. Two forms of crystallographically independent dimeric capsules were observed. As shown by the first form in Fig. 2, the two macrocycles adopt a bowl-shaped conformation and crossly overlay to each other through fourfold intermolecular C=O∙∙∙H–N hydrogen bonds (2.81–2.83 Å). The thiourea moieties lie in the bottom and adopt an unusual *syn-anti* configuration. The forming of two pairs of parallel intramolecular C=S∙∙∙H–N hydrogen bonds (3.39–3.40 Å) in the meantime helps lock the bowl-shaped macrocyclic conformation, leaving four *exo* NH sites for intermolecular hydrogen bonding. All the phenyl rings from different macrocycles stack onto each other, which may provide further stabilization.

**Fig. 3:**
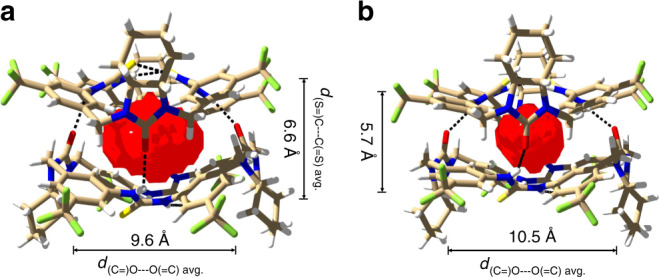
Cavity volume of the dimeric capsule M_2_. **a** Form 1 (72 Å^3^). **b** Form 2 (36 Å^3^). The cavity volume is determined by a spherical probe of 1.4 Å using SwissPdbViewer^54^ and depicted in red.

The buckling of the two bowl-shaped macrocycles produces a completely sealed dimeric capsule (Fig. 2c). The cavity volume was detected to be 72 Å^3^ for the first form (Fig. 3a). Surprisingly, while the overall interacting motif holds, the second form shows a much smaller cavity (36 Å^3^; Fig. 3b and Supplementary Table 4). The cavity reduction is due to flattening of the bowl-shaped macrocyclic conformation as reflected by shortened (S=)C---C(=S) and lengthened (C=)O---O(=C) dimensions (Fig. 3b vs 3a), suggesting considerable flexibility of the macrocyclic skeleton. The inter- and intramolecular hydrogen bonding, however, is not significantly affected. The existence of the two forms of dimeric capsules is probably caused by crystal packing effect, as the macrocyclic component has a rather flexible skeleton. In both forms, the solvent molecules were found too large to occupy the cavity.

### Dimeric assembly in solution

**Fig. 4:**
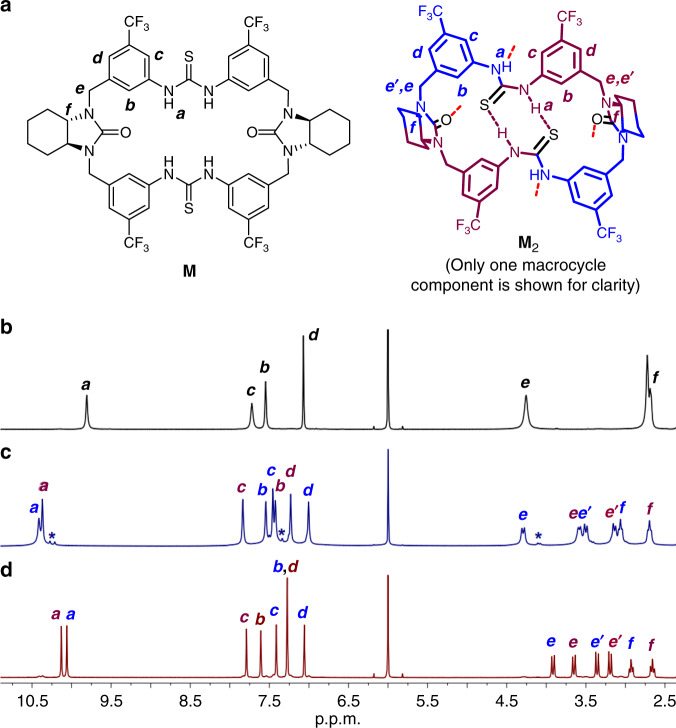
^1^H NMR (298 K, 500 M) of the macrocycle M in different solvent systems with or without xenon. **a** The chemical structures of the macrocycle and dimeric capsule with related protons labeled. **b** ^1^H NMR in (CDCl_2_)_2_/DMSO-*d*_*6*_ (5:1). The macrocycle exists as monomeric form. **c** ^1^H NMR in (CDCl_2_)_2_. The macrocycle exists as dimeric capsule form. Asterisk denotes the minor N_2_ inclusion peaks (for details see Supplementary Fig. 7). **d** ^1^H NMR in (CDCl_2_)_2_ after bubbling of xenon. The major peaks correspond to Xe ⊂  **M**_2_. In all cases, the initial concentration of the macrocycle ([**M**]_initial_) for preparing each solution is 10 mM.

The formation of dimeric assembly in solution was further studied. ^1^H NMR in (CDCl_2_)_2_/DMSO-*d*_*6*_ (5:1) shows a set of simple signals, consistent with an overall *D*_*2*_-symmetric free macrocycle structure in this competitive solvent system (Fig. 4b). In contrast, in pure (CDCl_2_)_2_, the spectrum totally changed to two sets of signals at equal intensity, in line with a reduced *C*_*2*_ macrocycle symmetry (Fig. 4c). The N*H* signals moved largely to downfield direction, suggesting favorable hydrogen bonding formation. By a combination of ^1^H, ^13^C and various 2D NMR (Supplementary Figs. 7–14), a dimeric capsular structure similar to that in crystal can be elucidated with all signals well assigned. The two sets of protons (in blue and purple) can exchange their positions through overturn of the *syn-anti* thiourea configuration as shown by 2D EXSY NMR (Supplementary Fig. 14; vide infra). As the solvent molecule is too large to fit, most capsules are likely to be empty except those occupied by N_2_ as indicated by the set of minor N_2_ inclusion peaks (Fig. 4c). High stability of the assembly was demonstrated by concentration-variable NMR as no essential spectral changes observed until dilution to 0.05 mM (Supplementary Fig. 23). A large dimerization constant of (1.9 ± 0.3) × 10^4^ M^−1^ was further determined by isothermal titration calorimetry (ITC; Supplementary Fig. 26). The dimeric assembly was also confirmed with high-resolution CSI-MS where a peak at *m*/*z* = 2111.5522 corresponding to [**M**_2_−H]^−^ dominated (Supplementary Fig. 27).

### Xenon binding

**Fig. 5:**
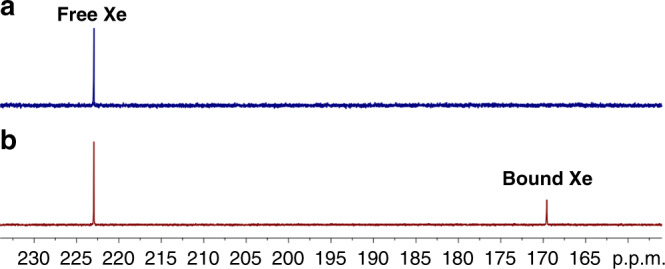
^129^Xe NMR (298 K, 138 MHz) of xenon saturated in (CDCl_2_)_2_ without or with the dimeric capsule. **a** The blank xenon solution. **b** In the presence of the dimeric capsule **M**_2_ (20 mM). The chemical shift of the free xenon was referenced according to the literature^22^.

The macrocycle can congruously form a stable, sealed dimeric capsule in both solid state and solution with a cavity possibly suited for xenon (*d*_w_ = 4.3 Å, *V* = 42 Å^3^). To our delight, through bubbling xenon gas, the original ^1^H NMR signals of the free dimeric capsule almost disappeared, instead a new group of sharp and well-resolved signals dominated, which can correspond to Xe ⊂ **M**_2_ (Fig. 4d). All signals were clearly assigned by a combination of ^1^H, ^13^C, and various 2D NMR (Supplementary Figs. 15–22). The upfield shift of N*H* signals and drastic changes of other proton signals may reflect a shielding effect of large electron cloud of xenon and an induced structural change due to tight inclusion (vide infra). The remarkable spectral difference is distinguished from the usually reported, relatively rigid hosts, suggesting the dimeric capsule is rather soft (as also shown in crystal) and can adaptively include the incoming xenon guest. The ^129^Xe NMR showed a similar slow exchange process and the signal of the bound xenon appeared at 169.6 ppm, largely upfield-shifted comparing to the free xenon in solution (Δ*δ* = −53.4 ppm; Fig. 5). A binding constant of 99 ± 4 M^−1^ was determined for xenon by ^1^H NMR integration (see Supplementary Methods and Supplementary Fig. 15). To the best of our knowledge, this is the highest xenon affinity reported to date for assembled hosts. As compared, a previous only known binding constant of 16 M^−1^ was reported for a metal-assembled Fe_4_L_6_ host^48^.

**Fig. 6:**
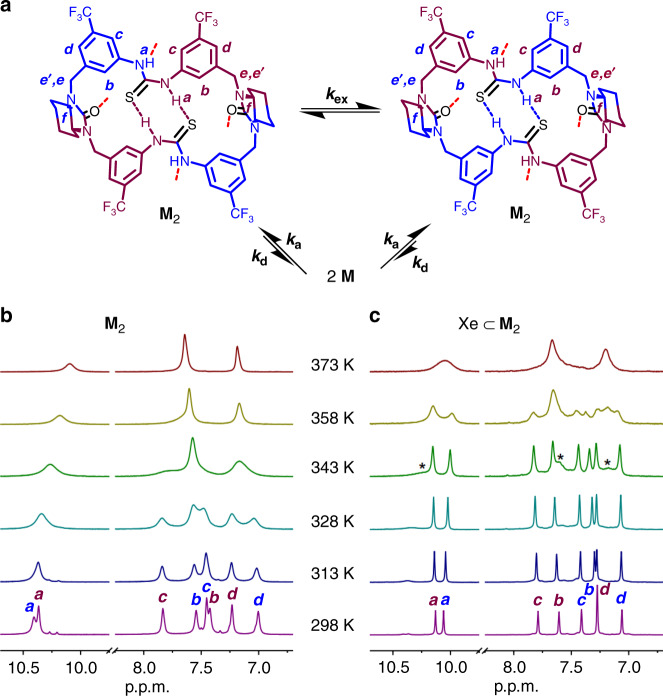
Determination of the xenon binding dynamics. **a** Proposed exchange mechanism of the two sets of macrocycle protons (depicted in blue and purple) in the dimeric capsule. **b**, **c** Partial temperature-variable ^1^H NMR (500 MHz, (CDCl_2_)_2_) of **M**_2_ and Xe ⊂ **M**_2_ respectively. Asterisk denotes the emerging peaks of free **M**_2_. In both cases, [**M**]_initial_ = 10 mM. For a complete set of spectra, see Supplementary Figs. 24 and 25.

The existence of two sets of position-exchangeable protons in the macrocyclic skeleton provides a unique probe for exploring the adaptive xenon binding dynamics. As intracapsular, simultaneous overturn of the many locked *syn-anti* configurated thiourea motifs seems unlikely, the position exchange is probably accomplished through a disassembly-reassembly pathway, i.e., once disassembled the conformation of the free macrocycle can be readily converted (Fig. 6a). Accordingly, the position-exchange rate can reflect the assembly-disassembly dynamics. The influence of xenon binding can thus be probed by comparing the temperature-variable ^1^H NMR before and after xenon inclusion (Fig. 6b, c). Upon temperature increasing, the two sets of signals started to coalesce and Xe ⊂ **M**_2_ showed an obviously higher coalescence temperature *T*_c_ and accordingly a higher activation energy Δ*G*^*‡*^ than **M**_2_ (17.4 ± 0.1 vs 16.4 ± 0.2 kcal mol^−1^; Supplementary Figs. 24 and 25 and Supplementary Table 6). This suggests upon xenon inclusion, the dimeric assembly becomes more kinetically stable; in other words, xenon binding could have induced a tighter capsular structure by fitting the soft macrocyclic skeletons. It is worth noting that even at elevated temperatures (up to 373 K), the assembly was still kept intact, once again showing its high thermodynamic stability.

### Structures of Xe ⊂ M_2_

**Fig. 7:**
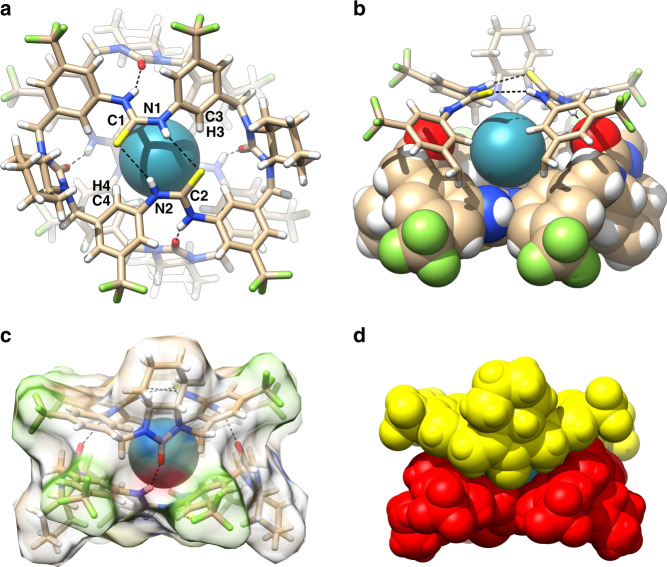
Crystal structure of Xe ⊂ M_2_. **a** Top view with the atoms contacting to xenon labeled (only the upside is labeled). **b** Side view with the front urea moiety cut off for clarity. **c** Side view with the macrocycle molecular surfaces shown. **d** Space filling view.

Two different high-quality crystals of Xe ⊂ **M**_2_ were independently obtained by diffusion of *n*-hexane to a macrocycle solution in chloroform or toluene saturated with xenon (Supplementary Tables 2–5). The first crystal crystallized in orthorhombic system as the free dimeric capsule with similar cell parameters (vide supra). As shown by the first form of the two crystallographically independent complexes (Fig. 7), the xenon was completely sealed in the cavity with a perfect packing coefficient of 52%^51^. In order to fit xenon, the cavity volume was expanded to 81 Å^3^. The xenon atom shows multiple contacts with the surrounding macrocyclic skeletons, including with the four *endo* nitrogen (3.83–3.87 Å; the sum of van der Waals radii of nitrogen and xenon is 3.71 Å^52^) and four carbon atoms (3.98–4.01 Å; the sum of van der Waals radii of carbon and xenon is 3.86 Å^52^) from the thiourea groups, and with the interior carbon (3.86–4.02 Å) and hydrogen atoms (3.23–3.42 Å; the sum of van der Waals radii of hydrogen and xenon is 3.36 Å^52^) from four diagonal phenyl rings (Fig. 7a). These contacts, especially that with polar nitrogen atoms, are different to those rigid aromatic faces or sole C–H sites in the usually reported xenon hosts. The second form showed an overall similar structure with a cavity of 69 Å^3^ and xenon packing coefficient of 61% (Supplementary Tables 4 and 5). The cavity size is largely expanded comparing to that free form of 36 Å^3^ (vide supra), reflecting the adaptive ability of this tight yet soft capsule. Again, the adaptation is accomplished through the adjust of the bowl-shaped macrocyclic conformation, rather than the change of hydrogen bonding distances.

The second crystal crystallized in a different monoclinic system, but exhibited a similar capsular structure and xenon binding motif (Supplementary Tables 4 and 5). The contacts of xenon to the relevant thiourea nitrogen atoms are even shorter (3.69–3.82 Å). It is worth noting that, even both crystals were grown under 1 atm xenon pressure, the averaged xenon occupancy in the cavity reaches 0.85 and 0.94 respectively, suggesting favored binding^53^. The sealed but reversible assembly capsule system could thus have potentials for sequestration and separation of xenon (see Supplementary Note 1 for detailed discussion).

In conclusion, a chiral bisurea-bisthiourea macrocycle was efficiently synthesized. This macrocycle can form stable dimeric capsular assembly in both solid state and solution. The capsule possesses a tight, sealed cavity, and effectively binds xenon. Different from other rigid hosts, the capsule is rather soft and can adaptively include xenon through induced fit of the surrounding macrocyclic skeletons. Multiple contacts including several unusual contacts to polar nitrogen atoms are engaged. The adaptive binding dynamic was demonstrated not only in crystal, but also in solution by taking the unique spectral features of the dimeric assembly. These knowledges should have advanced our current understanding on the host–guest chemistry of xenon. The sealed but reversible assembly could also have potentials for developing host materials toward xenon sequestration and separation. With the easy synthesis, modification, robust yet adjustable assembly features, further optimization of the xenon affinity, and ^129^Xe NMR related parameters should be feasible toward biosensing applications. The enantiopure form of the chiral assembly could also have advantages on chirality-sensitive detection.

## Supplementary information

Supplementary Information

## Data Availability

The X-ray crystallographic coordinates for structures reported in this study have been deposited at the Cambridge Crystallographic Data Centre (CCDC), under deposition numbers 2006631-2006633. These data can be obtained free of charge from The Cambridge Crystallographic Data Centre via www.ccdc.cam.ac.uk/data_request/cif. Supplementary methods for synthesis and characterization, crystallography, NMR, ITC studies, and additional data supporting the findings of this study are available in Supplementary Information file.
