# Supplementary material for: Xenon binding by a tight yet adaptive chiral soft capsule
Source: Nat Commun. 2020 Dec 7;11:6257. doi: 10.1038/s41467-020-20081-8 (PMC7721739; doi:10.1038/s41467-020-20081-8)
Supplement: Supplementary file 1 — Supplementary Information [file 41467_2020_20081_MOESM1_ESM.pdf]

Supplementary Information

Xenon Binding by a Tight yet Adaptive Chiral Soft Capsule

Shi-Xin Nie,[†,‡,‖] Hao Guo,[†,‡,‖] Teng-Yu Huang,[†,‡] Yu-Fei Ao,[†] De-Xian Wang,[†,‡] and Qi-Qiang Wang*,[†,‡]

[†]*Beijing National Laboratory for Molecular Sciences, CAS Key Laboratory of Molecular Recognition and Function, Institute of Chemistry, Chinese Academy of Sciences, Beijing 100190 (China)*

[‡]*University of Chinese Academy of Sciences, Beijing 100049 (China)*

[‖]*These two authors contributed equally to this work.*

qiqiangw@iccas.ac.cn

Supplementary Methods

General information. All chemicals were obtained from commercial sources and used without further purification unless stated otherwise. Anhydrous solvents such as acetone and dichloromethane were obtained by conventional methods with certain drying agents (distilling with CaH_2 for dichloromethane; molecular sieve for acetone). ^{1}H NMR and ^{13}C NMR spectra were recorded on Bruker 400 and 500 MHz NMR spectrometer. ^{1}H-^{1}H COSY, HSQC, HMBC, NOESY and 2D-EXSY spectra were recorded on Bruker 500 MHz NMR spectrometer. Chemical shifts are reported in ppm and referenced to tetramethylsilane or the residual solvent resonance. ^{129}Xe NMR spectra were recorded on a Bruker Avance III 500WB spectrometer (nominal frequency for ^{129}Xe =138.3 MHz) using a 5 mm BBFO probe (temperature = 298 K). The chemical shift of free xenon in $(CDCl_2)_2$ was referenced to the resonance frequency of pure xenon gas extrapolated to zero pressure according to literature.[1] Mass spectra were obtained on LCMS-2010, Shimadzu Co. (for CSI). Infrared spectra were recorded on JASCO-4800 (for compounds characterization) and Nicolet-6700 FT-IR spectrometer. Elemental analysis was recorded on Carlo Erba 1106. Melting points are uncorrected.

Synthesis and characterization.

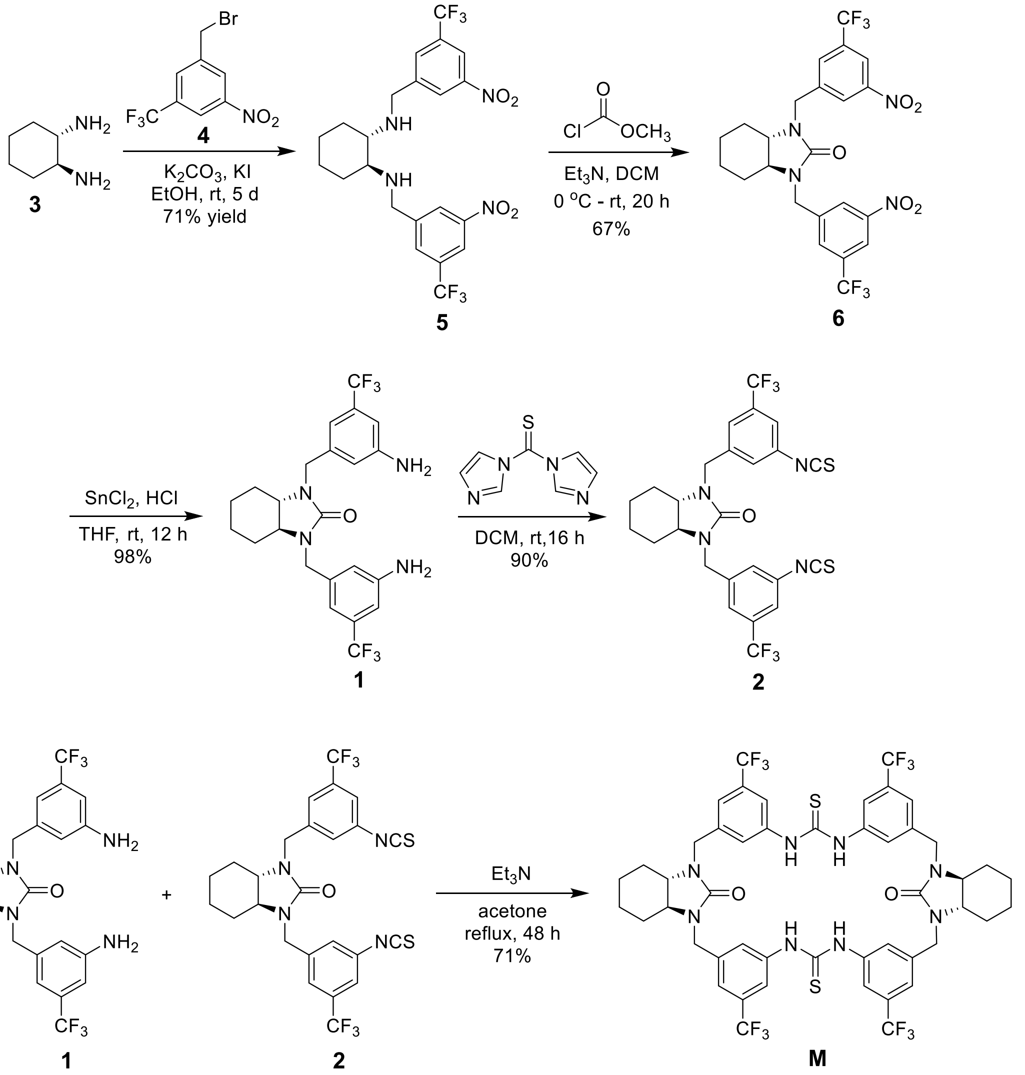

(1*S*,2*S*)-*N*[1],*N*[2]-bis(3-nitro-5-(trifluoromethyl)benzyl)cyclohexane-1,2-diamine (5): To a solution of (1*S*,2*S*)-N^1,N^2-dimethylcyclohexane-1,2-diamine **3** (1.14 g, 10 mmol) in ethanol (50 mL) was added K_2CO_3 (5.25 g, 38 mmol), KI (220 mg) and 1-(bromomethyl)-3-nitro-5-(trifluoromethyl)benzene **4** (5.96 g, 21 mmol). The mixture was stirred at room temperature for 5 d, and then the solvent was evaporated under reduced pressure. Water (50 mL) was then added, and the mixture was extracted with ethyl acetate (50 mL × 3). The organic layers were combined and dried over Na_2SO_4, then the solvent was removed under reduced pressure. The residue was subjected to column chromatography on silica gel (petroleum ether/ethyl acetate = 1:1) to give **5** as a yellow oil (3.70 g, yield: 71%).

^{1}H NMR ($CDCl_3$, 400 MHz) δ (ppm) 8.42 (s, 2H), 8.33 (s, 2H), 7.96 (s, 2H), 4.09

(d, J = 14.5 Hz, 2H), 3.92 (d, J = 14.5 Hz, 2H), 2.36-2.29 (m, 2H), 2.19-2.15 (m, 2H), 2.04 (br, s, 2H), 1.79-1.76 (m, 2H), 1.30-1.25 (m, 2H), 1.12- 1.04 (m, 2H); ^{13}C NMR ($CDCl_3$, 100 MHz) δ (ppm) 148.6, 145.3, 132.4 (q, J = 33.8 Hz, *C*-CF_3), 130.5 (q, J = 3.7 Hz, *C*-C-CF_3), 126.0, 123.0, (q, J = 271.3 Hz, *C*F_3), 119.4 (q, J = 3.8 Hz, *C*-C-CF_3), 61.7, 50.1, 31.8, 24.9; IR (KBr) ν 3303, 3097, 2931, 2857, 1541, 1469, 1448, 1325, 1174, 1134 cm^{-1}; HRMS (ESI^+) calc. for $[M+H]^+$ ($C_{22}H_{23}F_6N_4O_4^+$), 521.1618, found 521.1616; $[\alpha]_D^{25}$ = +31.2° (c = 0.6, $CHCl_3$).

(3a*S*,7a*S*)-1,3-bis(3-nitro-5-(trifluoromethyl)benzyl)octahydro-*2H*-benzo[*d*]imidazol-2-one (6): Under an argon atmosphere, to a solution of **5** (9.88 g, 19 mmol) in anhydrous dichloromethane (80 mL) at 0 °C was added triethylamine (5.8 mL, 28.5 mmol) and methyl chloroformate (2.4 mL, 30.4 mmol). The resulting mixture was stirred at room temperature for 20 h. The solvent was evaporated under reduced pressure and the residue was subjected to column chromatography on silica gel (petroleum ether/ethyl acetate = 2:1) to give **6** as a white solid (7.03 g, yield: 67%).

Mp 90-91 °C; ^{1}H NMR ($CDCl_3$, 400 MHz) δ (ppm) 8.41 (s, 2H), 8.38 (s, 2H), 7.93 (s, 2H), 4.86 (d, J = 16.0 Hz, 2H), 4.28 (d, J = 16.0 Hz, 2H), 2.95-2.93 (m, 2H), 1.82-1.81 (m, 4H), 1.32-1.24 (m, 4H); ^{13}C NMR ($CDCl_3$, 100 MHz) δ (ppm) 163.5, 148.7, 142.5, 132.8 (q, J = 34.1 Hz, *C*-CF_3), 130.3 (q, J = 3.4 Hz, *C*-C-CF_3), 122.9 (q, J = 271.3 Hz, *C*F_3), 120.0 (q, J = 3.9 Hz, *C*-C-CF_3), 63.4, 46.94, 28.9, 24.1; IR (KBr) ν 3097, 2941, 2869, 1706, 1542, 1325, 1133 cm^{-1}; HRMS ($APCI^+$) calc. for $[M+H]^+$ ($C_{23}H_{21}F_6N_4O_5^+$), 547.1411, found 547.1405; $[\alpha]_D^{25}$ = -4.4° (c = 0.5, $CHCl_3$).

(3a*S*,7a*S*)-1,3-bis(3-amino-5-(trifluoromethyl)benzyl)octahydro-*2H*-benzo[*d*]imidazol-2-one (1): To a solution of **6** (546 mg, 1 mmol) in THF (50 mL) was added a solution of $SnCl_2·2H_2O$ (1.50 g, 6.4 mmol) in *conc.* hydrochloric acid (2.5 mL). The mixture was stirred at room temperature for 12 h. Then a solution of 40% aq. NaOH was added to reach pH > 10, and the organic layer was separated. The aqueous layer was extracted with ethyl acetate (20 mL× 3). The combined organic layers were dried over Na_2SO_4, and then evaporated under reduced pressure. The residue was subjected

to column chromatography on silica gel (dichloromethane/methanol = 10:1) to give **1** as a white solid (481 mg, yield: 98%).

Mp 240 °C (decomposition); ^{1}H NMR (DMSO-d_6, 300 MHz) δ (ppm) 6.73 (s, 4H), 6.69 (s, 2H), 5.59 (s, br, 4H), 4.29-4.17 (m, 4H), 2.70-2.68 (m, 2H), 1.85-1.83 (m, 2H), 1.68-1.66 (m, 2H), 1.23-1.09 (m, 4H); ^{13}C NMR (DMSO-d_6, 75 MHz) δ (ppm) 162.7, 149.5, 140.1, 129.8 (q, J = 30.5 Hz, C-CF_3), 124.4 (q, J = 270.5 Hz, CF_3), 116.3, 110.84, 110.80, 108.4, 61.5, 46.0, 28.0, 23.6; IR (KBr) ν 3348, 3227, 2940, 2867, 1684, 1628, 1374, 1259, 1170, 1117 cm^{-1}; HRMS (ESI$^+$) calc. for $[M+Na]^+$ ($C_{23}H_{24}F_6N_4ONa^+$), 509.1747, found 509.1744; $[\alpha]_D^{25}$ = +2.0° (c = 0.5, acetone).

(3a*S*,7a*S*)-1,3-bis(3-isothiocyanato-5-(trifluoromethyl)benzyl)octahydro-*2H*-benzo[*d*]imidazol-2-one (2): To a solution of **1** (243 mg, 0.5 mmol) in anhydrous dichloromethane (20 mL) was added 1,1'-thiocarbonyldiimidazole (356 mg, 2 mmol). The mixture was stirred at room temperature for 16 h. The solvent was evaporated under reduced pressure and the residue was subjected to column chromatography on silica gel (petroleum ether/ethyl acetate = 3:1) to give **2** as a yellow solid (257 mg, yield: 90%).

Mp 88-89 °C; ^{1}H NMR ($CDCl_3$, 400 MHz) δ (ppm) 7.46 (s, 2H), 7.38 (s, 2H), 7.36 (s, 2H), 4.66 (d, J = 15.8 Hz, 2H), 4.21 (d, J = 15.8 Hz, 2H), 2.84-2.82 (m, 2H), 1.80-1.78 (m, 4H), 1.29-1.21 (m, 4H); ^{13}C NMR ($CDCl_3$, 100 MHz) δ (ppm) 163.4, 141.8, 138.0, 133.1, 132.8, 132.6 (q, J = 33.0 Hz, C-CF_3), 128.0, 123.1 (q, J = 3.7 Hz, C-C-CF_3), 123.1 (q, J = 271.3 Hz, CF_3), 121.8 (q, J = 3.7 Hz, C-C-CF_3), 63.0, 46.8, 28.7, 24.1; IR (KBr) ν 3230, 2939, 2866, 2083, 1700, 1606, 1464, 1359, 1341, 1242, 1126 cm^{-1}; HRMS (APCI$^+$) calc. for $[M+H]^+$ ($C_{25}H_{21}F_6N_4OS_2^+$), 571.1056, found 571.1050; $[\alpha]_D^{25}$ = +11.6° (c = 0.5, $CHCl_3$).

Macrocycle M: Under an argon atmosphere, to a solution of **1** (2.14 g, 4.4 mmol) in anhydrous acetone (100 mL) was added **2** (2.53 g, 4.4 mmol) and triethylamine (1.53 mL, 11 mmol). The resulting mixture was reflux for 48 h. The solvent was evaporated under reduced pressure and the residue was subjected to column chromatography on silica gel (petroleum ether/ethyl acetate = 2:1) to give **M** as a white solid (3.28 g, yield:

71%).

Mp 186-187 °C; ^{1}H NMR ($(CDCl_2)_2$/DMSO-d_6 (5:1), 500 MHz) δ (ppm) 9.81 (s, 4H), 7.72 (s, 4H), 7.55 (s, 4H), 7.07 (s, 4H), 4.25 (s, 4H), 2.69-2.67 (m, 4H), 1.91-1.79 (m, 4H), 1.61-1.59 (m, 4H), 1.52-1.51 (m, 4H), 1.06-1.02 (m, 8H); ^{13}C NMR ($(CDCl_2)_2$ /DMSO-d_6 (5:1), 125 MHz) δ (ppm) 178.1, 161.9, 138.6, 129.1 (q, J = 31.8 Hz, C-CF_3), 125.0, 122.7 (q, J = 271.1 Hz, CF_3), 119.3, 117.9, 60.6, 44.7, 27.0, 22.9; IR (KBr) ν 3197, 2940, 2867, 1695, 1538, 1464, 1339, 1262, 1175, 1131 cm^{-1}; HRMS (ESI$^+$) calc. for $[M+Na]^+$ ($C_{48}H_{44}F_{12}N_8O_2S_2Na^+$), 1079.2729, found 1079.2734; Anal. Calcd. for $C_{48}H_{44}F_{12}N_8O_2S_2$: C,54.54; H, 4.20; N, 10.60. Found: C,54.22; H, 4.20; N, 10.44; $[\alpha]_D^{25}$ = -148.4° (c = 0.5, $CHCl_3$).

Crystallography studies. The single crystal of the empty dimeric capsule $\mathbf{M}_2$ was cultivated by diffusion of *n*-hexane to a solution of the macrocycle **M** in toluene. For xenon complex, two kinds of single crystals were obtained in different conditions. The first single crystal $[Xe \subset \mathbf{M}_2](C_6H_{14})_{0.5}$ was cultivated by diffusion of *n*-hexane to a solution of the macrocycle **M** in chloroform saturated with xenon and kept under an atmosphere of xenon by connection to a xenon balloon. The second crystal $[Xe \subset \mathbf{M}_2]$ was cultivated by diffusion of *n*-hexane to a solution of the macrocycle **M** in toluene saturated with xenon and kept under an atmosphere of xenon by connection to a xenon balloon.

Single crystal X-ray diffraction data were collected on a XtaLAB Synergy R, HyPix diffractometer for the structures using CuKα radition (λ = 1.54184 Å) at 170 K. The intensity data were collected by the omega scans techniques, scaled, and reduced with CrysAlisPro 1.171.40.67a (Rigaku OD, 2019). X-rays were provided by a fine-focus sealed X-ray tube operated at 40 kV and 30 mA. Lattice constants were determined with the CrysAlisPro 1.171.40.67a (Rigaku OD, 2019) using peak centers for 27089 reflections ($\mathbf{M}_2$), 26996 reflections ($[Xe \subset \mathbf{M}_2](C_6H_{14})_{0.5}$, recrystallized from chloroform/*n*-hexane) and 27145 reflections ($[Xe \subset \mathbf{M}_2]$), recrystallized from toluene/*n*-hexane). Integrated reflection intensities were produced and the correction of the collected intensities for absorption was done using the CrysAlisPro 1.171.40.67a (Rigaku OD, 2019) program. Using Olex2 (Dolomanov, 2009), the structures were solved with the ShelXT (Sheldrick, 2015) structure solution program using Intrinsic Phasing and refined with the ShelXL (Sheldrick, 2015) refinement package using Least Squares minimisation. All non-hydrogen atoms were refined anisotropically, and hydrogen atoms attached to carbon atoms were fixed at their ideal positions. For the included xenon atom, the refining mode of the occupancy factor was set as free, so that an exact occupancy factor of xenon can be obtained. See Supplementary Tables 1-5 for crystal data, structure refinement and related structural parameters.

Supplementary Table 1. Crystal data and structure refinement for $\mathbf{M}_2$

	$\mathbf{M}_2$
CCDC no.	2006631
Empirical formula	$C_{96}H_{88}F_{24}N_{16}O_4S_4$
Formula weight	2114.06
Temperature	170.00(10) K
Wavelength	1.54184 Å
Crystal system	Orthorhombic
Space group	$P2_12_12$
a	25.4810(2) Å
b	15.26260(10) Å
c	34.7335(3) Å
α	90°
β	90°
γ	90°
Volume	13508.08(18) $Å^3$
Z	4
Density (calculated)	1.040 Mg/m^3
Absorption coefficient	1.318 mm^{-1}
F(000)	4352
Crystal size	0.23 x 0.15 x 0.13 mm^3
Theta range for data collection	2.544 to 75.559°
Index ranges	-32<=h<=31, -19<=k<=14, -43<=l<=42
Reflections collected	124806
Independent reflections	27085 [R(int) = 0.0422]
Completeness to theta = 67.684°	100.0 %
Absorption correction	Semi-empirical from equivalents
Max. and min. transmission	1.00000 and 0.59548
Refinement method	Full-matrix least-squares on F^2
Data / restraints / parameters	27085 / 3 / 1297
Goodness-of-fit on F^2	1.057
Final R indices [I>2sigma(I)]	R1 = 0.0585, wR2 = 0.1665
R indices (all data)	R1 = 0.0687, wR2 = 0.1804
Absolute structure parameter	0.033(4)
Extinction coefficient	n/a
Largest diff. peak and hole	0.676 and -0.326 e.$Å^{-3}$

Supplementary Table 2. Crystal data and structure refinement for [Xe⊂**M**$_2$](C_6H_{14})$_{0.5}$ (recrystallized from chloroform/*n*-hexane).

	[Xe⊂**M**$_2$](C_6H_{14})$_{0.5}$
CCDC no.	2006632
Empirical formula	$C_{99}H_{95}F_{24}N_{16}O_4S_4Xe_{0.85}$
Formula weight	2269.08
Temperature	169.99(10) K
Wavelength	1.54184 Å
Crystal system	Orthorhombic
Space group	$P2_12_12$
a	15.20650(10) Å
b	25.5576(2) Å
c	34.7160(3) Å
α	90°
β	90°
γ	90°
Volume	13492.08(18) Å^3
Z	4
Density (calculated)	1.117 Mg/m^3
Absorption coefficient	2.975 mm^{-1}
F(000)	4636
Crystal size	0.61 x 0.45 x 0.332 mm^3
Theta range for data collection	2.546 to 75.533°
Index ranges	-18<=h<=19, -31<=k<=31, -43<=l<=42
Reflections collected	126438
Independent reflections	26996 [R(int) = 0.0459]
Completeness to theta = 67.684°	99.9 %
Absorption correction	Semi-empirical from equivalents
Max. and min. transmission	1.00000 and 0.39329
Refinement method	Full-matrix least-squares on F^2
Data / restraints / parameters	26996 / 232 / 1393
Goodness-of-fit on F^2	1.026
Final R indices [I>2sigma(I)]	R1 = 0.0478, wR2 = 0.1310
R indices (all data)	R1 = 0.0540, wR2 = 0.1387
Absolute structure parameter	-0.0083(17)
Extinction coefficient	n/a
Largest diff. peak and hole	0.870 and -0.661 e.Å^{-3}

Supplementary Table 3. Crystal data and structure refinement for [Xe ⊂ **M**$_2$] (recrystallized from toluene/*n*-hexane).

	[Xe ⊂ **M**$_2$]
CCDC no.	2006633
Empirical formula	$C_{96}H_{88}F_{24}N_{16}O_4S_4Xe_{0.94}$
Formula weight	2237.81
Temperature	169.99(10) K
Wavelength	1.54178 Å
Crystal system	Monoclinic
Space group	C 1 2 1
a	38.4103(16) Å
b	15.2649(7) Å
c	26.3033(9) Å
α	90°
β	117.824(4)°
γ	90°
Volume	13639.3(11) Å^3
Z	4
Density (calculated)	1.090 Mg/m^3
Absorption coefficient	3.107 mm^{-1}
F(000)	4556
Crystal size	0.21 x 0.132 x 0.114 mm^3
Theta range for data collection	2.400 to 76.273°
Index ranges	-48<=h<=47, -18<=k<=19, -32<=l<=32
Reflections collected	122198
Independent reflections	27145 [R(int) = 0.0930]
Completeness to theta = 67.679°	100.0%
Absorption correction	Semi-empirical from equivalents
Max. and min. transmission	1.00000 and 0.23192
Refinement method	Full-matrix least-squares on F^2
Data / restraints / parameters	27145 / 682 / 1373
Goodness-of-fit on F^2	1.080
Final R indices [I>2sigma(I)]	R1 = 0.0725, wR2 = 0.2164
R indices (all data)	R1 = 0.0919, wR2 = 0.2384
Absolute structure parameter	0.005(4)
Extinction coefficient	n/a
Largest diff. peak and hole	0.791 and -0.354 e.Å^{-3}

Supplementary Table 4. Structural parameters of the dimeric capsule for free $\mathbf{M}_2$ and Xe⊂$\mathbf{M}_2$.

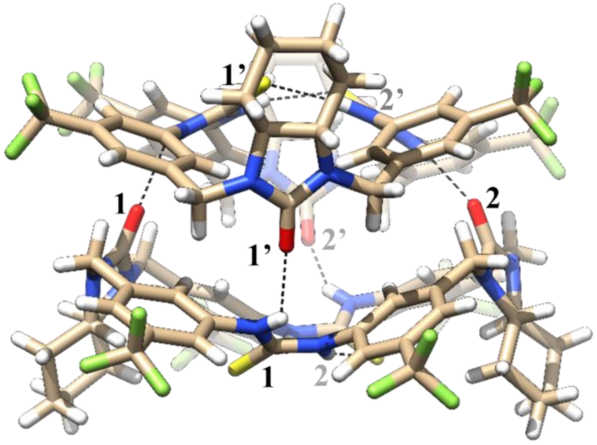

Structure		Xe occupancy	V_{guest} (Å^3)[a]	V_{cavity} (Å^3)[b]	PC[c]	C=O···H-N (intermolecular) (Å)				C=S···H-N (intramolecular) (Å)				(S=)C---C(=S) (Å)	
						C=O 1, 2		C=O 1', 2'		C=S 1, 2		C=S 1', 2'		1-1'	2-2'
**M**$_2$	Form 1	-	-	72	-	2.808	2.832	2.808	2.832	3.393	3.399	3.393	3.399	6.338	6.875
	Form 2	-	-	36	-	2.906	2.867	2.906	2,867	3.333	3.354	3.333	3.354	5.623	5.721
[Xe⊂$\mathbf{M}_2$](C_6H_{14})$_{0.5}$ (recrystallized from chloroform/*n*-hexane)	Form 1	86%	42	81	52%	2.817	2.863	2.816	2.863	3.396	3.409	3.396	3.409	6.464	6.969
	Form 2	85%	42	69	61%	2.872	2.881	2.872	2.881	3.356	3.373	3.356	3.373	6.240	6.334
[Xe⊂$\mathbf{M}_2$] (recrystallized from toluene/*n*-hexane)	Form 1	100%	42	82	51%	2.782	2.783	2.782	2.783	3.376	3.406	3.376	3.406	6.717	6.717
	Form 2	89%	42	73	58%	2.824	2.824	2.843	2.843	3.379	3.379	3.332	3.332	6.410	6.410

[a]van der Waals volume of the guest. [b]Cavity volume determined by a spherical probe of 1.4 Å using Swiss PdbViewer software. [c]Packing coefficients.

Supplementary Table 5. Close contacts between xenon and the dimeric capsule in Xe$\subset$**M**$_2$.

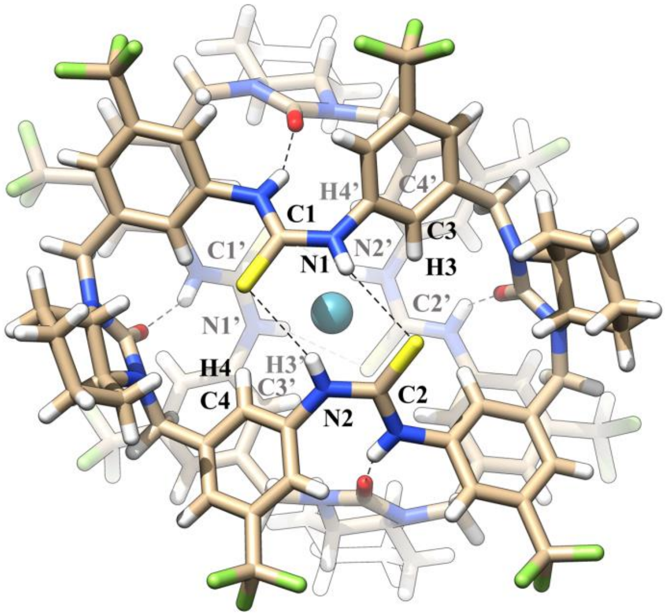

Structure		(C=S-)N···Xe (Å)				(S=)C···Xe (Å)				(Ph)H···Xe (Å)				(Ph)C···Xe (Å)			
		N1, N2		N1', N2'		C1, C2		C1', C2'		H3, H4		H3', H4'		C3, C4		C3', C4'	
[Xe$\subset$**M**$_2$](C_6H_{14})$_{0.5}$ (recrystallized from chloroform/*n*-hexane)	Form 1	3.834	3.872	3.834	3.872	4.010	3.983	4.010	3.983	3.233	3.415	3.233	3.415	3.855	4.021	3.855	4.021
	Form 2	3.653	3.711	3.653	3.711	3.792	3.783	3.792	3.783	3.378	3.461	3.378	3.461	3.943	4.022	3.943	4.022
[Xe$\subset$**M**$_2$] (recrystallized from toluene/*n*-hexane)	Form 1	3.768	3.816	3.768	3.816	3.972	3.992	3.972	3.992	3.350	3.328	3.350	3.328	3.952	3.914	3.952	3.914
	Form 2	3.691	3.691	3.706	3.706	3.827	3.827	3.832	3.832	3.457	3.457	3.414	3.414	3.979	3.979	3.980	3.980

NMR studies of M. The spectra of the monomeric macrocycle **M** were obtained in $(CDCl_2)_2$/DMSO-d_6 (5:1). A series of ^{1}H NMR, ^{13}C NMR, DEPT135, ^{1}H-^{1}H COSY, HSQC, HMBC spectra were collected and the assignment of all the signals can be readily achieved (Supplementary Figs. 1-6).

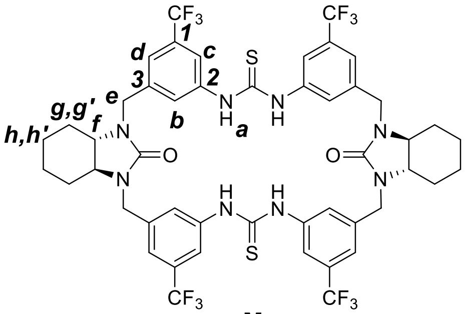

M

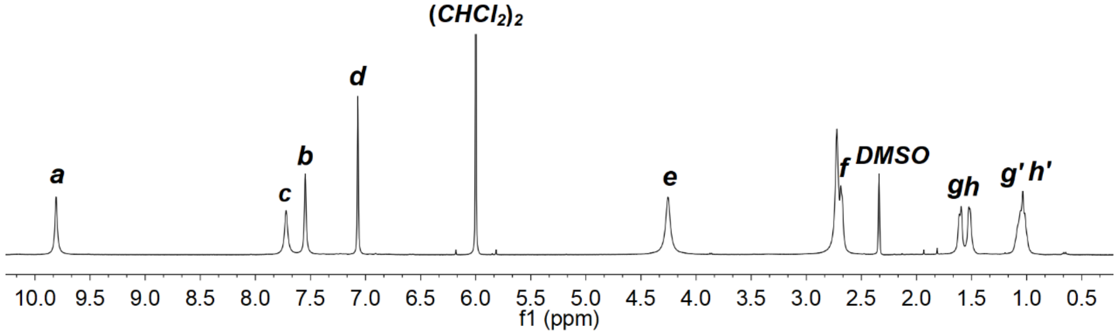

Supplementary Fig. 1 ^{1}H NMR (298 K, 500 MHz) of **M** in $(CDCl_2)_2$/DMSO-d_6 (5:1) ([**M**] = 10 mM).

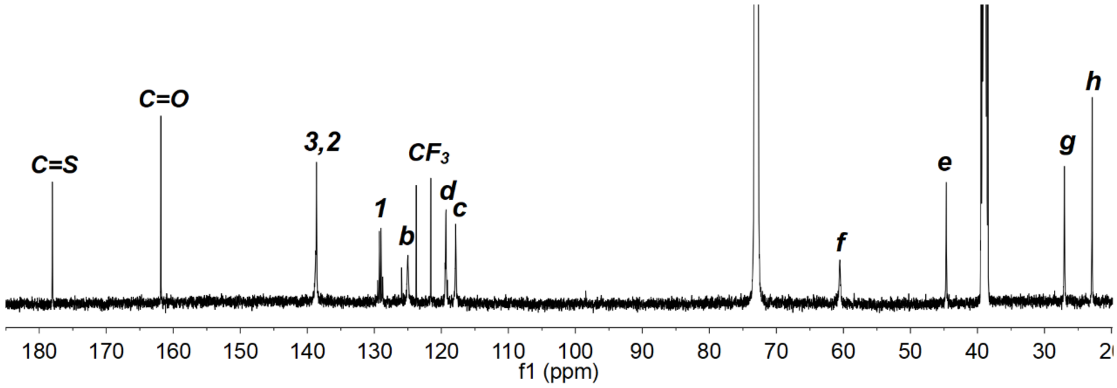

Supplementary Fig. 2 ^{13}C NMR (298 K, 125 MHz) of **M** in $(CDCl_2)_2$/DMSO-d_6 (5:1) ([**M**] = 10 mM).

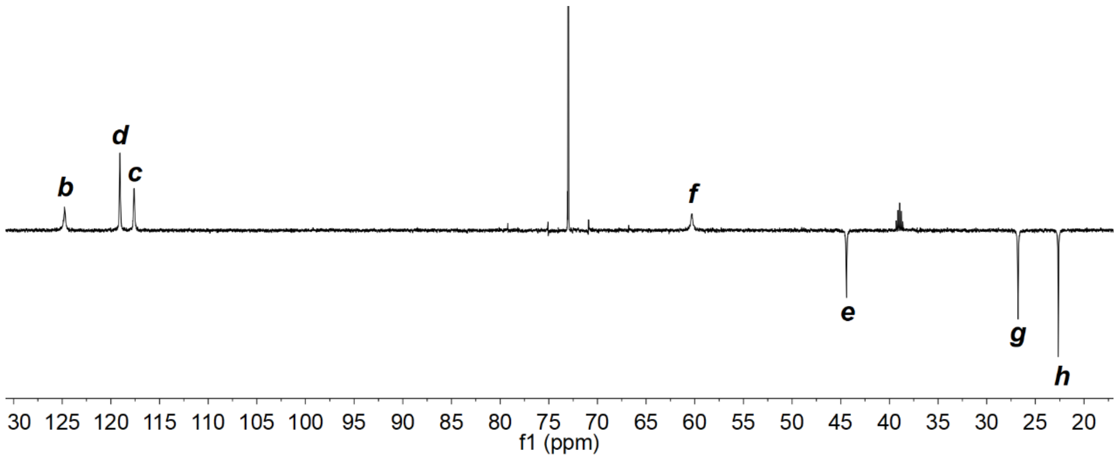

Supplementary Fig. 3 DEPT135 NMR (298 K, 500 MHz) of **M** in $(CDCl_2)_2$/DMSO-d_6 (5:1) ([**M**] = 10 mM).

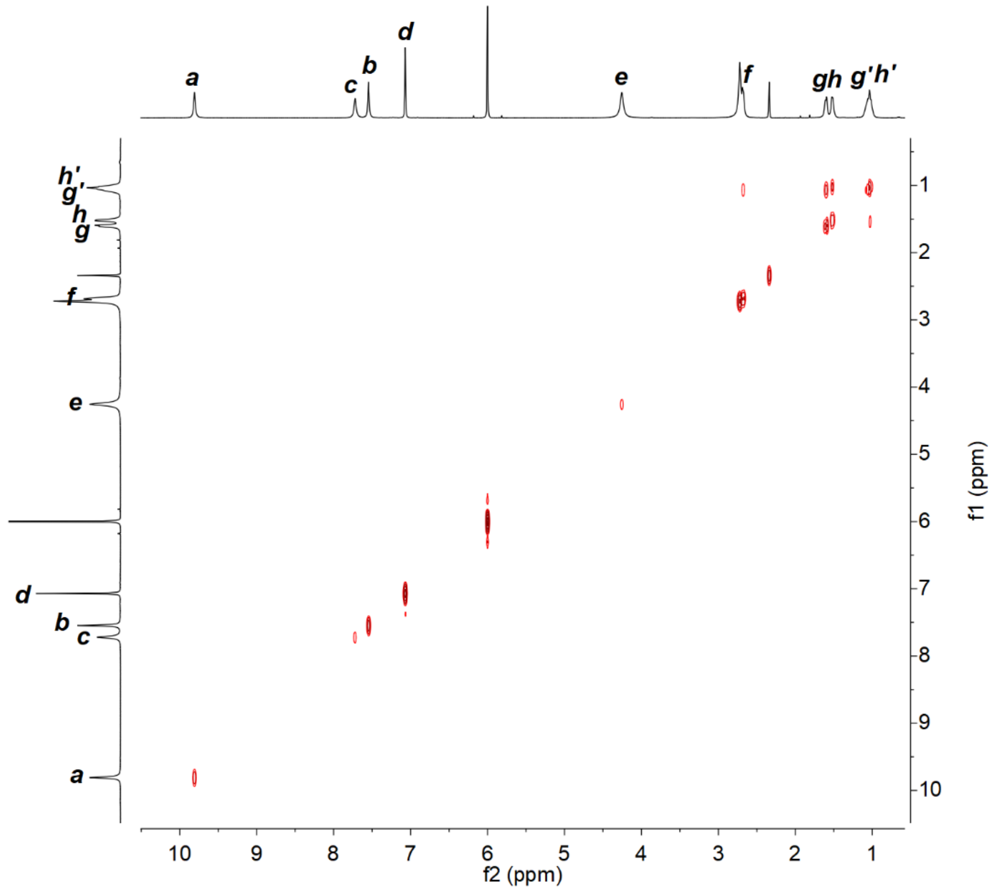

Supplementary Fig. 4 ^{1}H-^{1}H COSY NMR (298 K, 500 MHz) of **M** in $(CDCl_2)_2$/DMSO-d_6 (5:1) ([**M**] = 10 mM).

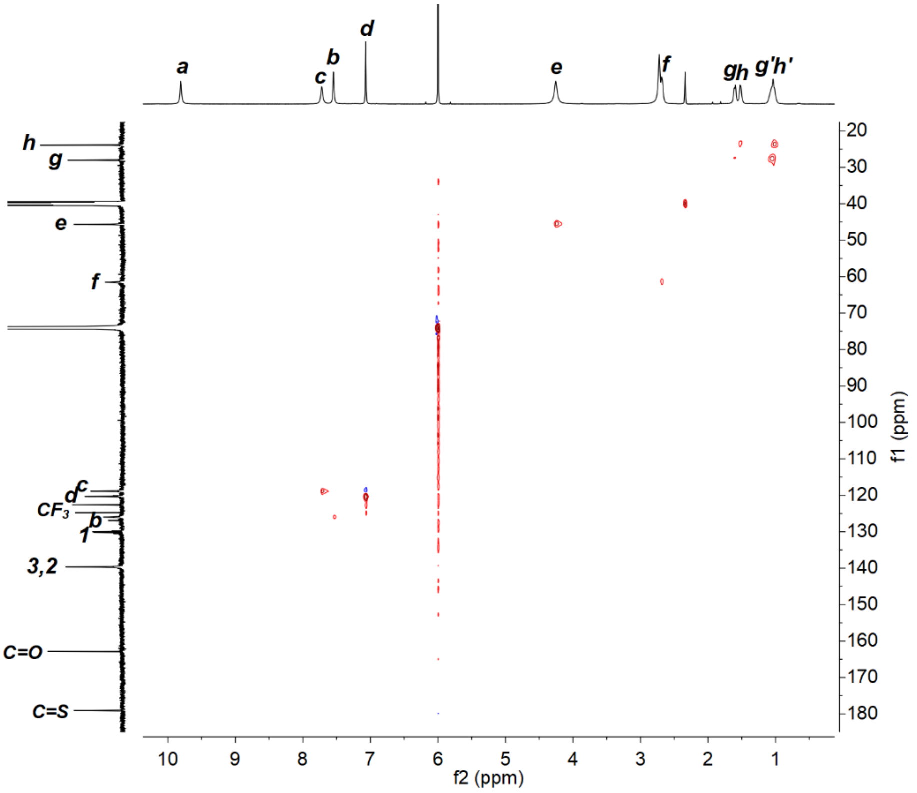

Supplementary Fig. 5 HSQC NMR (298 K, 500 MHz) of **M** in $(CDCl_2)_2$/DMSO-d_6 (5:1) ([**M**] = 10 mM).

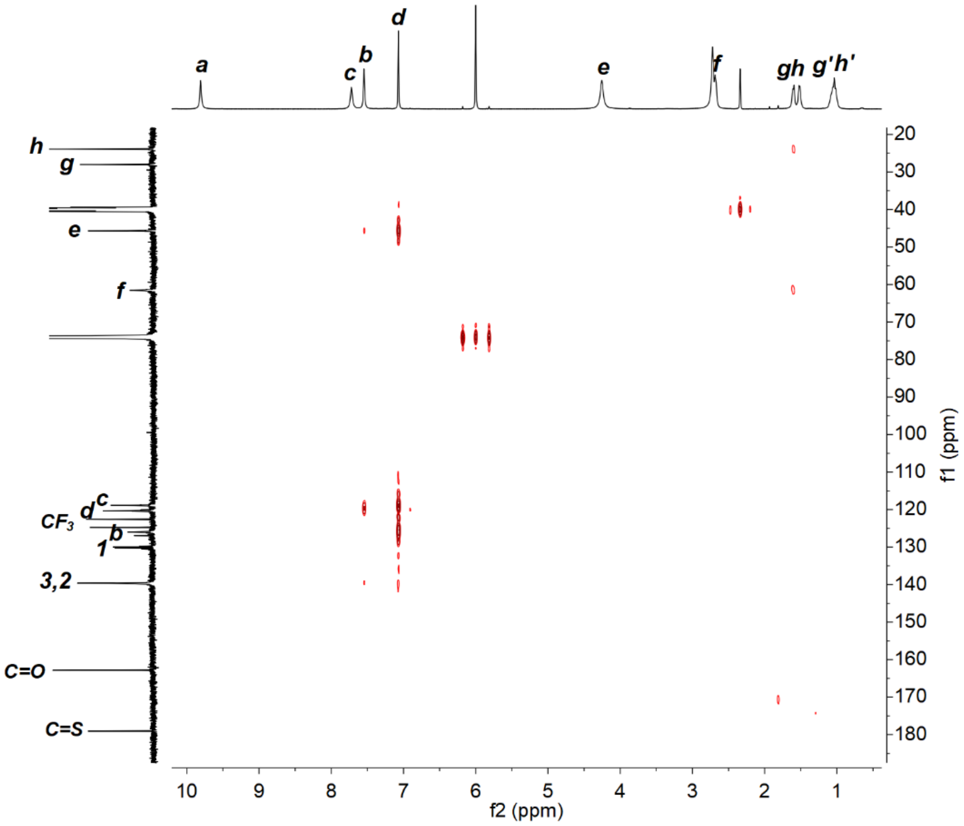

Supplementary Fig. 6 HMBC NMR (298 K, 500 MHz) of **M** in $(CDCl_2)_2$/DMSO-d_6 (5:1) ([**M**] = 10 mM).

NMR studies of the dimeric capsule M_2. The spectra of the empty dimeric capsule $\mathbf{M}_2$ were obtained in $(CDCl_2)_2$. A series of ^{1}H NMR, ^{13}C NMR, DEPT135, ^{1}H-^{1}H COSY, HSQC, HMBC, NOESY, and 2D EXSY spectra were collected and the assignment of all the signals can be achieved (Supplementary Figs. 7-14). Based on 2D EXSY spectrum, two-site position exchange signals were observed and the two divided sets of signals can thus be determined (in blue and purple, respectively). The assignments of all the signals in each set can be obtained by a combination of the above spectra. The identifying of which set is which was realized by the correlation signal observed between N*H*$_a$ and *C*=S in HMBC spectrum (no correlation peak was observed between N*H*$_a$ and *C*=S; this was compared to that the similar correlation peak was also observed in Xe $\subset$ $\mathbf{M}_2$, *vide infra*).

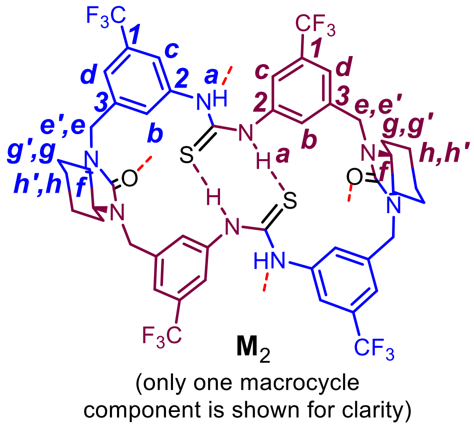

$\mathbf{M}_2$
(only one macrocycle component is shown for clarity)

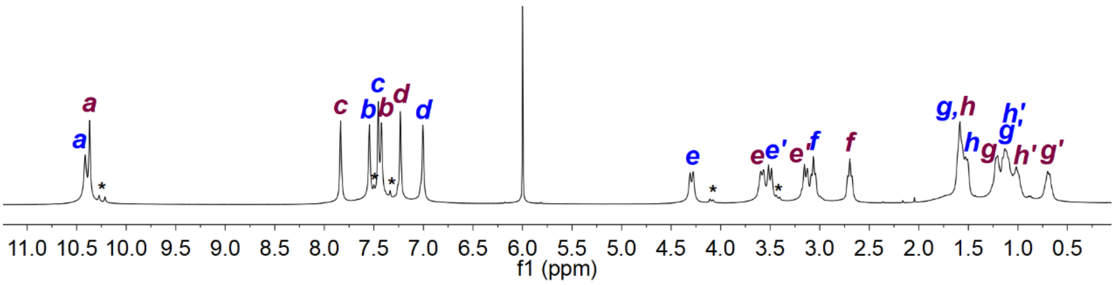

Supplementary Fig. 7 ^{1}H NMR (298 K, 500 MHz) of $\mathbf{M}_2$ in $(CDCl_2)_2$ ($[\mathbf{M}]_{initial}$ = 10 mM). "*" denotes the minor N_2 inclusion peaks. These minor peaks disappeared through bubbling of O_2 to the solution, and re-appeared after bubbling of N_2.

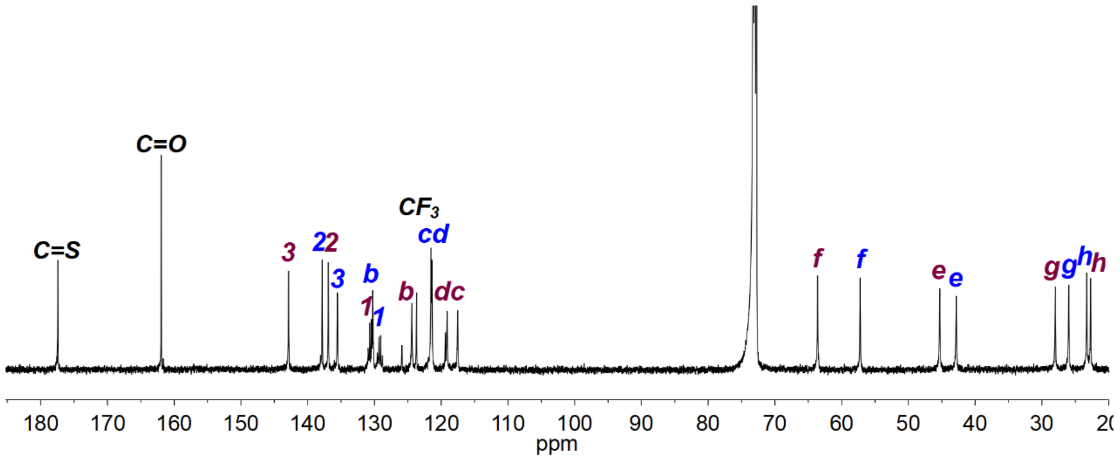

Supplementary Fig. 8 ^{13}C NMR (298 K, 125 MHz) of $\mathbf{M}_2$ in $(CDCl_2)_2$ ($[\mathbf{M}]_{initial}$ = 10 mM).

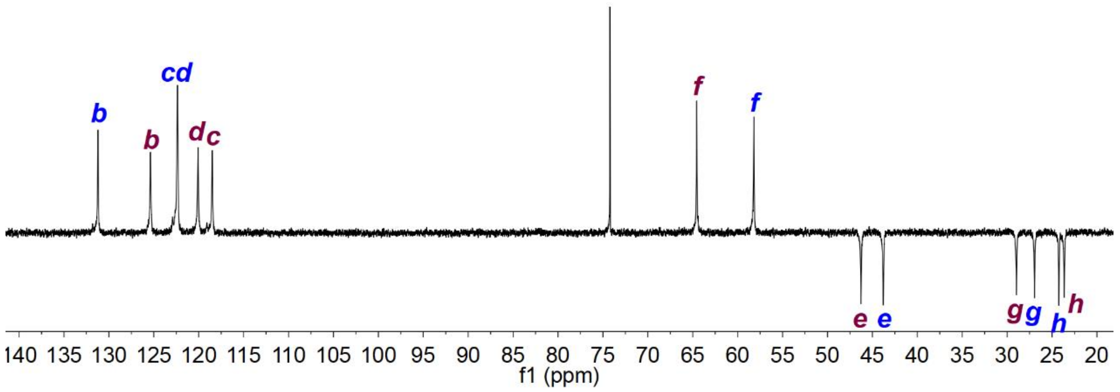

Supplementary Fig. 9 DEPT135 NMR (298 K, 500 MHz) of $\mathbf{M}_2$ in $(CDCl_2)_2$ ($[\mathbf{M}]_{initial}$ = 10 mM).

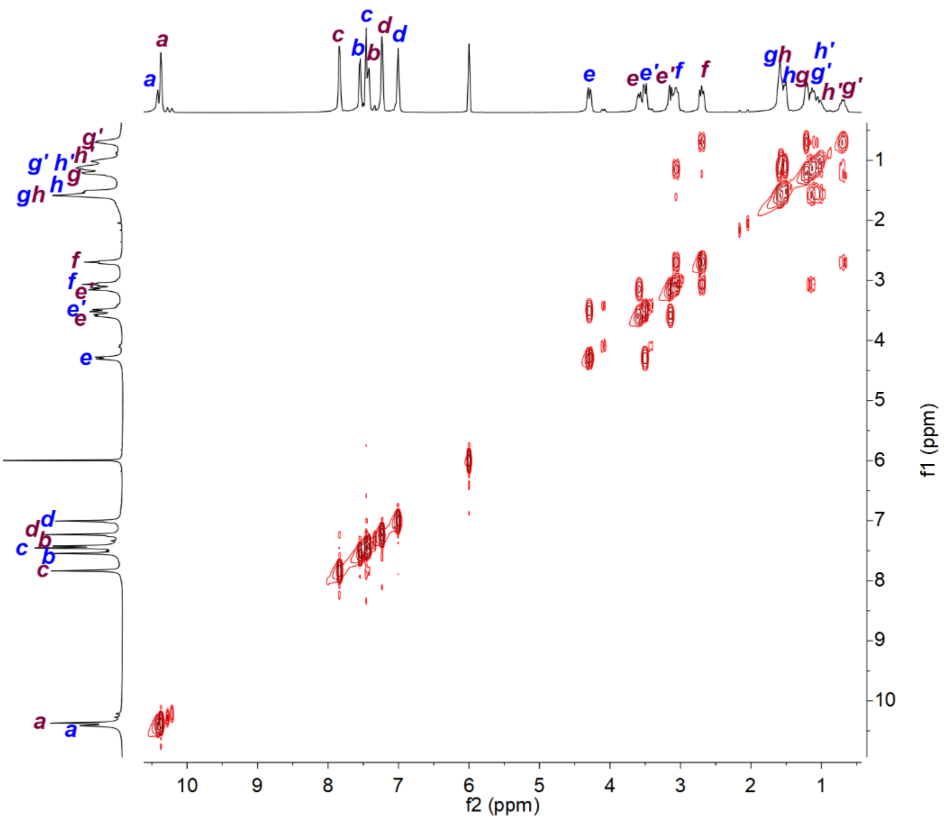

Supplementary Fig. 10 ^{1}H-^{1}H COSY NMR (298 K, 500 MHz) of $\mathbf{M}_2$ in $(CDCl_2)_2$ ($[\mathbf{M}]_{initial}$ = 10 mM).

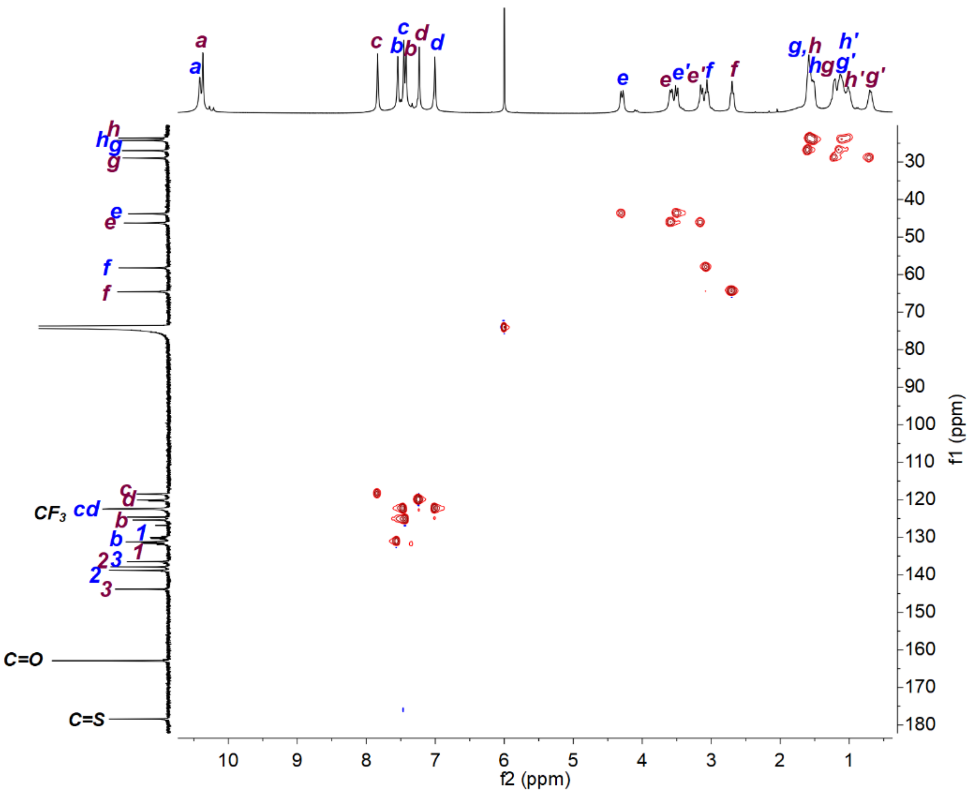

Supplementary Fig. 11 HSQC NMR (298 K, 500 MHz) of $\mathbf{M}_2$ in $(CDCl_2)_2$ ($[\mathbf{M}]_{initial}$ = 10 mM).

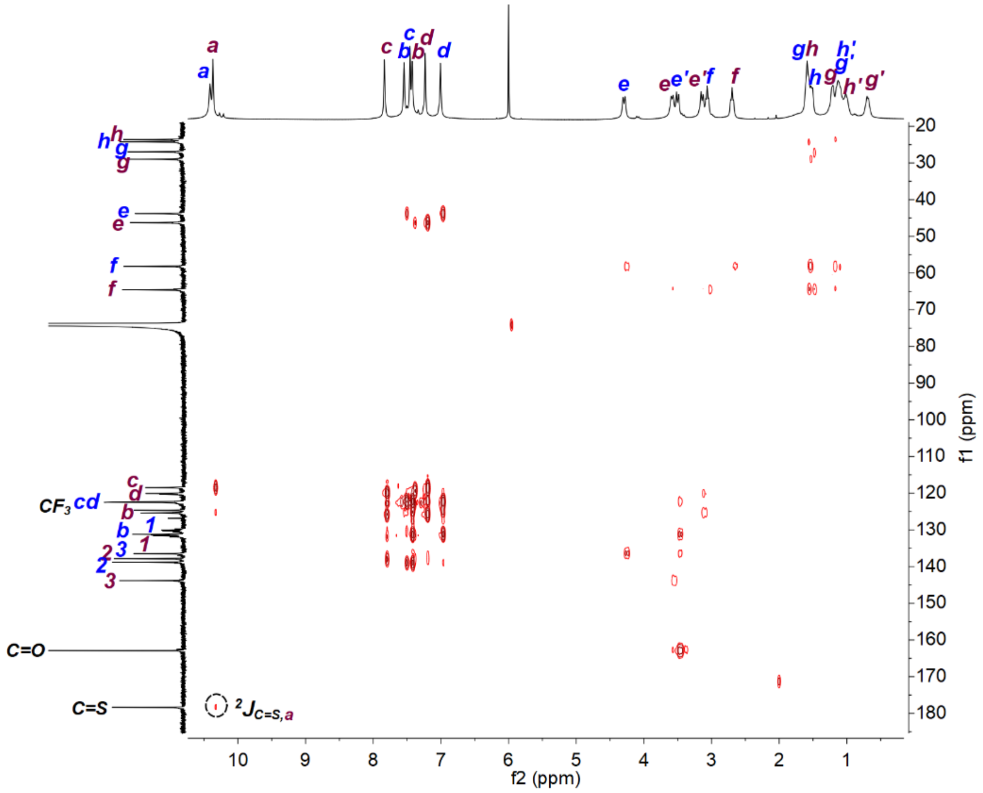

Supplementary Fig. 12 HMBC NMR (298 K, 500 MHz) of $\mathbf{M}_2$ in $(CDCl_2)_2$ ($[\mathbf{M}]_{initial}$ = 10 mM).

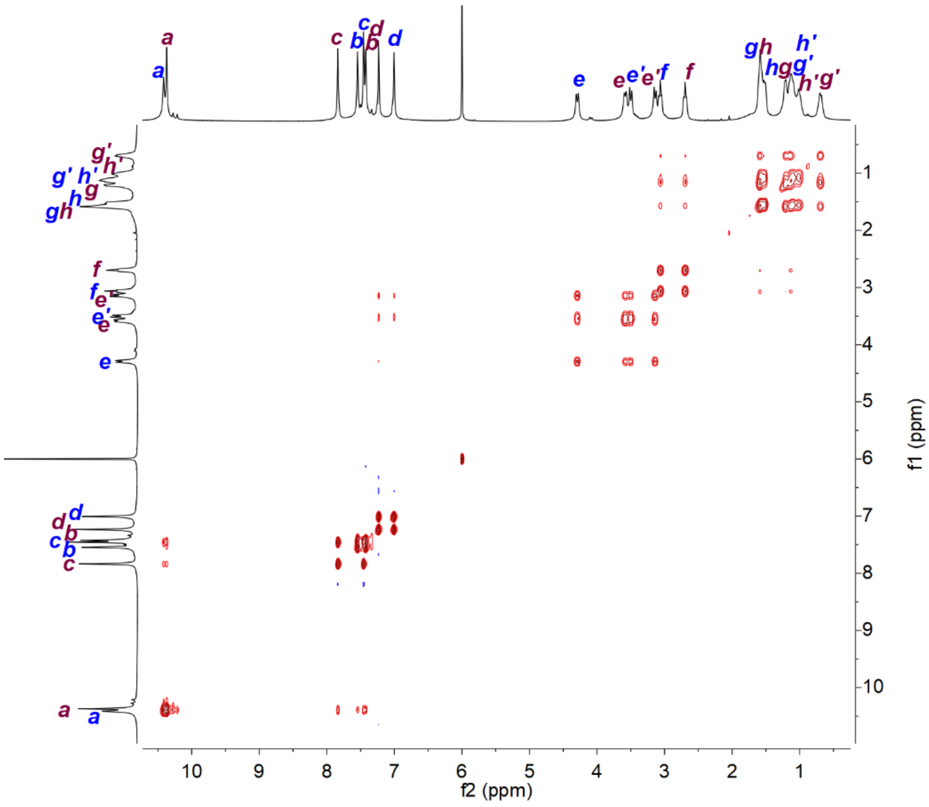

Supplementary Fig. 13 NOESY NMR (298 K, 500 MHz, mixing time = 0.3 s) of $\mathbf{M}_2$ in $(CDCl_2)_2$ ($[\mathbf{M}]_{initial}$ = 10 mM).

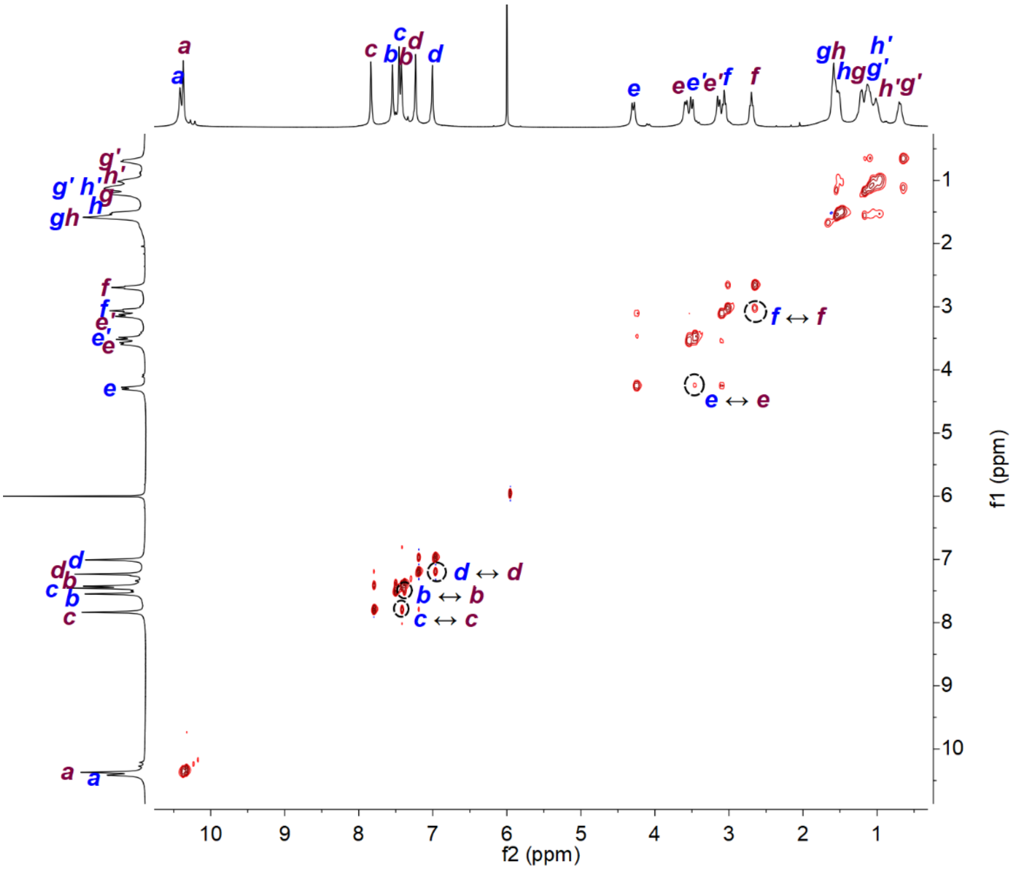

Supplementary Fig. 14 2D EXSY NMR (298 K, 500 MHz, mixing time = 0.05 s) of **M**$_2$ in $(CDCl_2)_2$ ([**M**]$_{initial}$ = 10 mM). The main exchange peaks between the two sets of signals were indicated.

NMR studies of Xe ⊂ M_2. The spectra of the Xe ⊂ $\mathbf{M}_2$ complex were obtained in $(CDCl_2)_2$ saturated with xenon. A series of ^{1}H NMR, ^{13}C NMR, DEPT135, ^{1}H-^{1}H COSY, HSQC, HMBC, NOESY, and 2D EXSY spectra were collected and the assignment of all the signals can be achieved (Supplementary Figs. 15-22). The assignments of the signals in each set (in blue or purple) can be obtained by a combination of the spectra. In the 2D EXSY spectrum, two-site position exchange signals were no longer observed (the xenon binding suppressed the exchange). The identifying of which set is which was realized by the NOE correlation signal observed between NH_a and H_c, H_c, and the correlation signal between NH_a and H_b in the NOESY spectrum. In the HMBC spectrum, a correlation signal between NH_a and *C*=S was observed, but no correlation peak was observed between NH_a and *C*=S (this also gave an indication for identifying the two sets of signals for $\mathbf{M}_2$, *vide supra*).

Determination of xenon binding constants. The binding constant of xenon with $\mathbf{M}_2$ was determined from ^{1}H NMR integration of the related species, including free $\mathbf{M}_2$, and Xe ⊂ $\mathbf{M}_2$. The concentration of the free xenon was estimated to be the xenon solubility in 1,1,2,2-tetrachloroethane (0.101 M).[2] The calculation was carried out from the below equation. Four independent experiments were performed and an averaged value of $K = 99 \pm 4$ M^{-1} was obtained.

$$K = \frac{[\mathrm{Xe} \subset \mathbf{M}_2]}{[\mathbf{M}_2]\times[\mathrm{Xe}]} = \frac{\mathrm{R}_{[\mathrm{Xe} \subset \mathbf{M}_2]}\times[\mathbf{M}_2]_{\mathrm{total}}}{(\mathrm{R}_{\mathbf{M}_2}\times[\mathbf{M}_2]_{\mathrm{total}})\times[\mathrm{Xe}]_{\mathrm{free}}} \quad \text{Supplementary Equation (1)}$$

$R_{[\mathrm{Xe} \subset \mathbf{M}_2]}$ and $R_{\mathbf{M}_2}$ are the integration ratios of the related species to the total dimer species respectively.

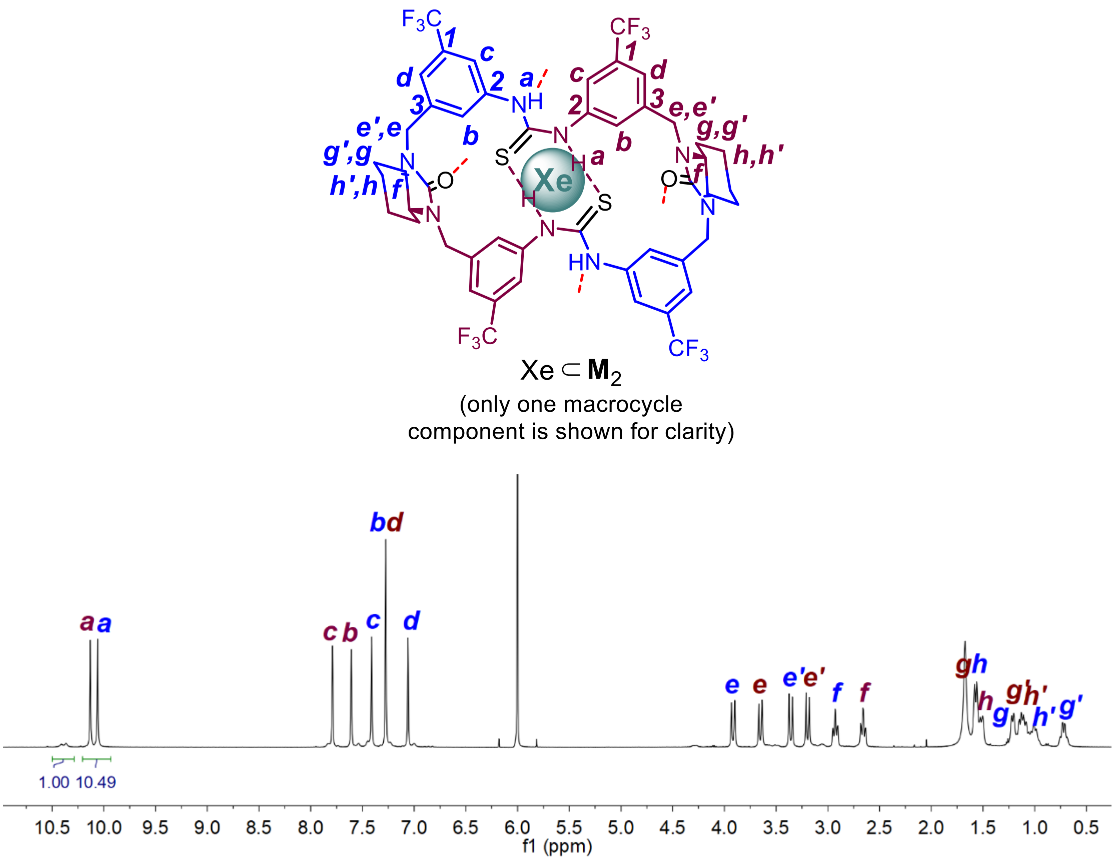

Supplementary Fig. 15 ^{1}H NMR (298 K, 500 MHz) of Xe⊂**M**$_2$ in $(CDCl_2)_2$ (obtained through bubbling of xenon to a solution of **M**$_2$) ([**M**]$_{initial}$ = 10 mM). The relevant integration of the free and bound dimeric capsule species for determination of the xenon binding constant was shown (the N*H* signals were applied).

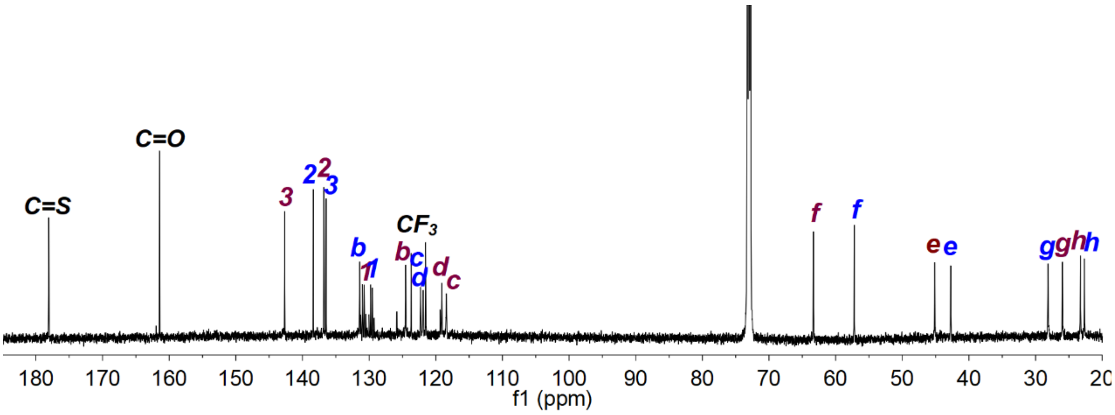

Supplementary Fig. 16 ^{13}C NMR (298 K, 125 MHz) of Xe⊂**M**$_2$ in $(CDCl_2)_2$ (obtained through bubbling of xenon to a solution of **M**$_2$) ([**M**]$_{initial}$ = 10 mM).

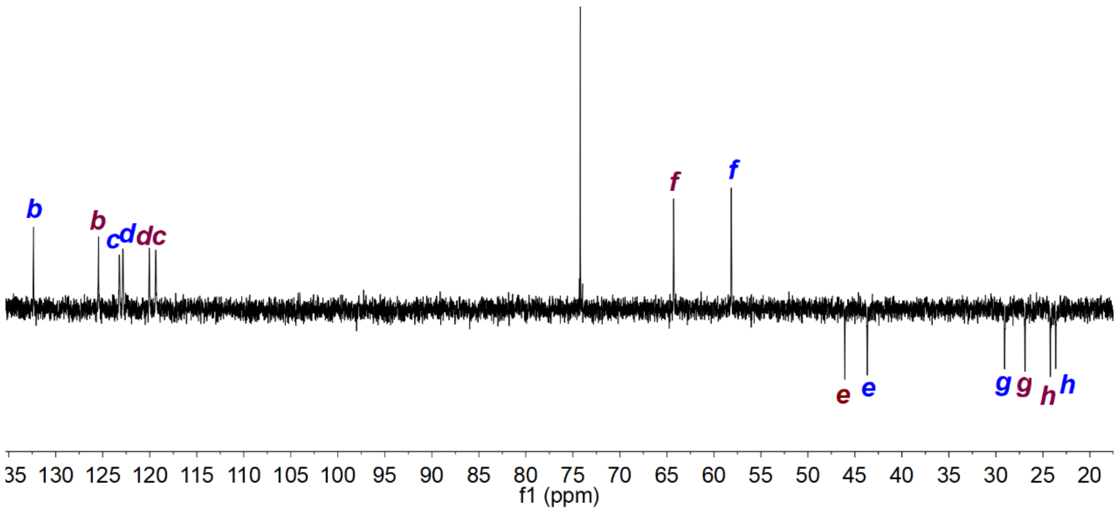

Supplementary Fig. 17 DEPT135 NMR (298 K, 500 MHz) of Xe $\subset$ **M**$_2$ in $(CDCl_2)_2$ (obtained through bubbling of xenon to a solution of **M**$_2$) ([**M**]$_{initial}$ = 10 mM).

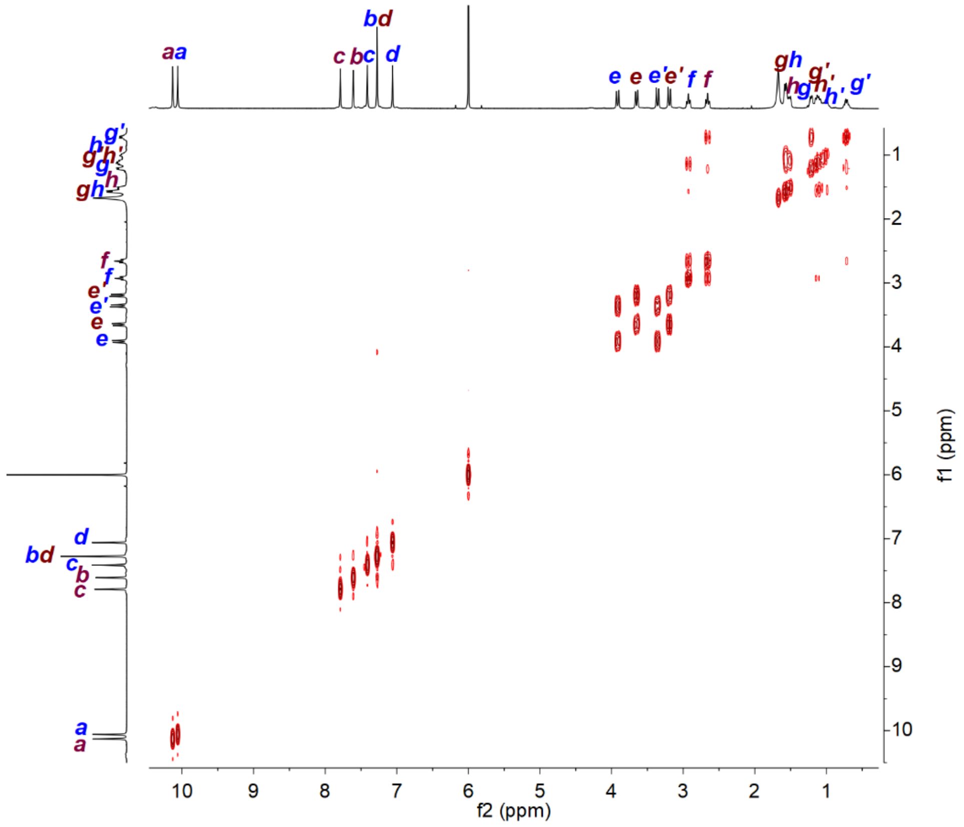

Supplementary Fig. 18 ^{1}H-^{1}H COSY NMR (298 K, 500 MHz) of Xe $\subset$ **M**$_2$ in $(CDCl_2)_2$ (obtained through bubbling of xenon to a solution of **M**$_2$) ([**M**]$_{initial}$ = 10 mM).

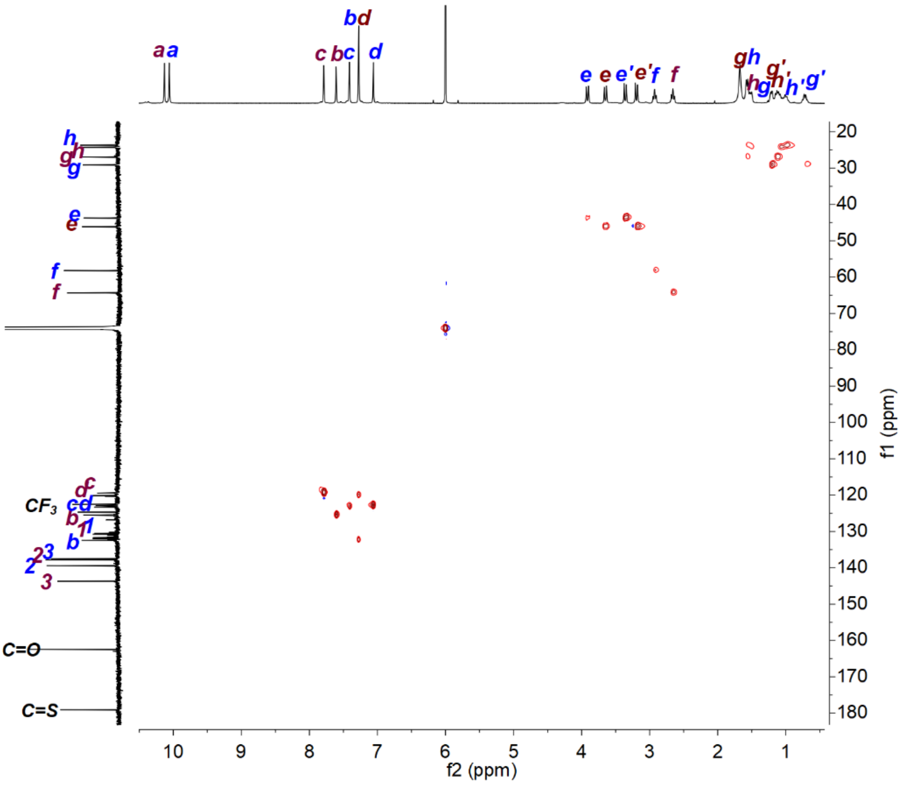

Supplementary Fig. 19 HSQC NMR (298 K, 500 MHz) of Xe ⊂ $\mathbf{M}_2$ in $(CDCl_2)_2$ (obtained through bubbling of xenon to a solution of $\mathbf{M}_2$) ($[\mathbf{M}]_{initial}$ = 10 mM).

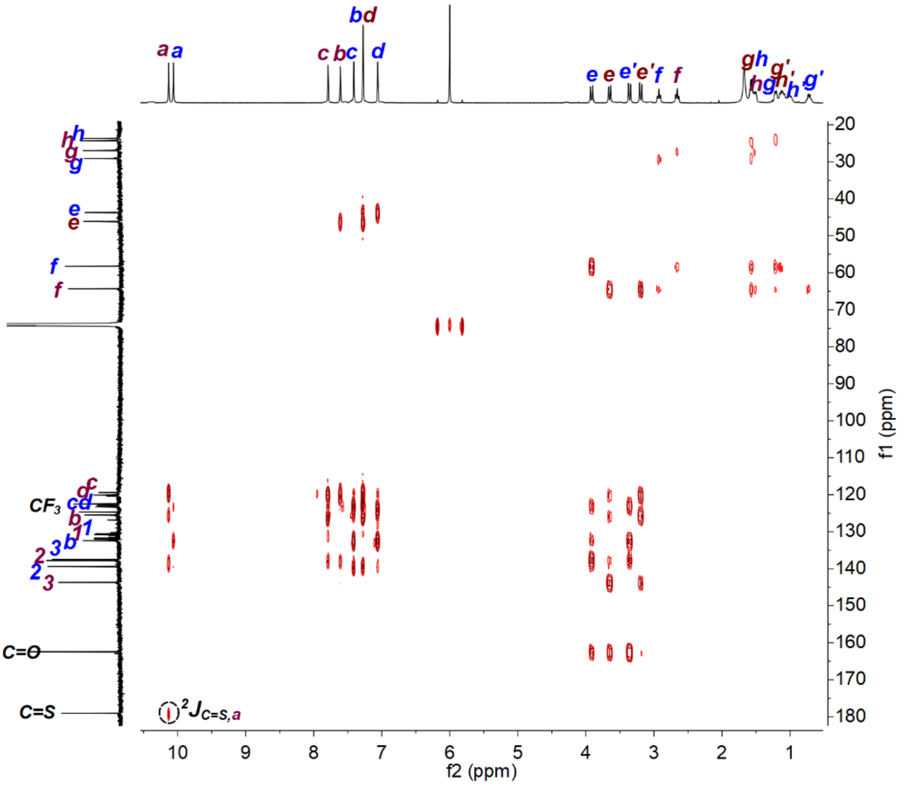

Supplementary Fig. 20 HMBC NMR (298 K, 500 MHz) of Xe ⊂ $\mathbf{M}_2$ in $(CDCl_2)_2$ (obtained through bubbling of xenon to a solution of $\mathbf{M}_2$) ($[\mathbf{M}]_{initial}$ = 10 mM).

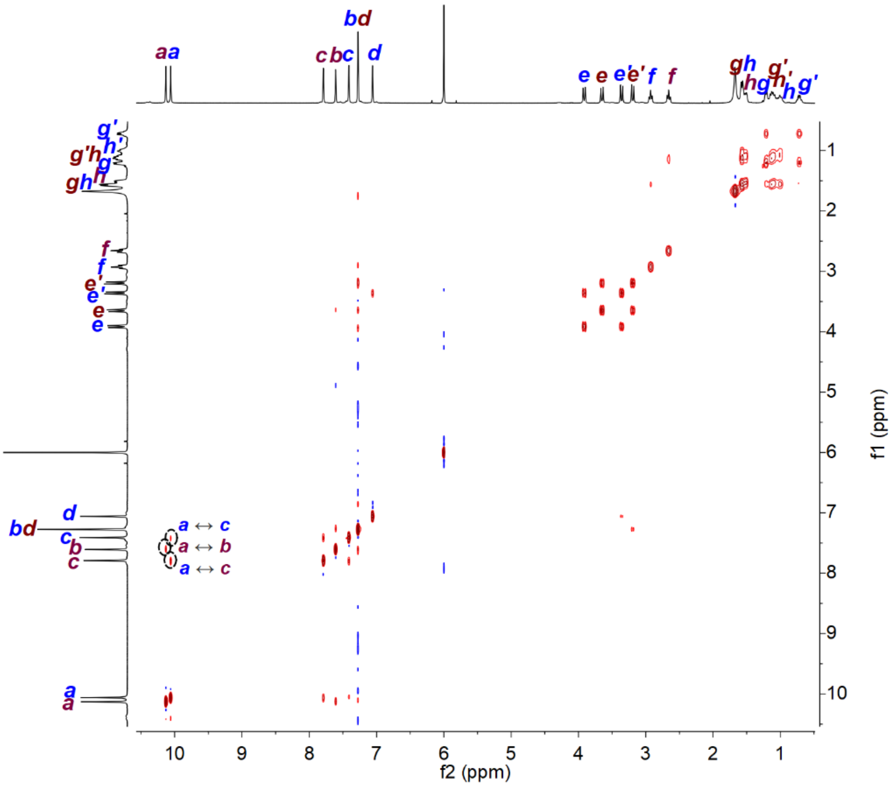

Supplementary Fig. 21 NOESY NMR (298 K, 500 MHz, mixing time = 0.5 s) of Xe $\subset$ **M**$_2$ in $(CDCl_2)_2$ (obtained through bubbling of xenon to a solution of **M**$_2$) ($[\mathbf{M}]_{initial}$ = 10 mM).

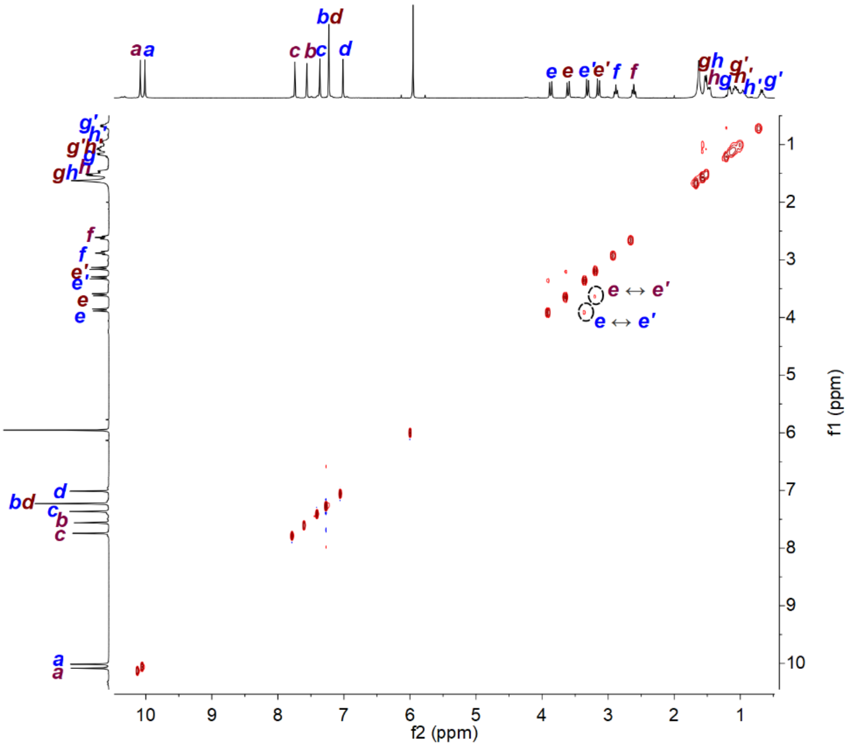

Supplementary Fig. 22 2D EXSY NMR (298 K, 500 MHz, mixing time = 0.05 s) of Xe $\subset$ **M**$_2$ in $(CDCl_2)_2$ (obtained through bubbling of xenon to a solution of **M**$_2$) ($[\mathbf{M}]_{initial}$ = 10 mM). Only the exchange signal between ***e*** and ***e’***, and that between ***e*** and ***e’*** were observed which could be caused by the inverting of the diastereotopic CH_2 protons.

Concentration-variable ¹H NMR studies. Concentration-variable ¹H NMR spectra of $\mathbf{M}_2$ were recorded in $(CDCl_2)_2$ at room temperature. The spectra of a series of solutions in different concentrations were recorded (Supplementary Fig. 23). In the tested concentration range as below as 0.05 mM, no signals corresponding to the free monomeric macrocycle were observed. This suggested a high thermodynamics stability of the dimeric capsule.

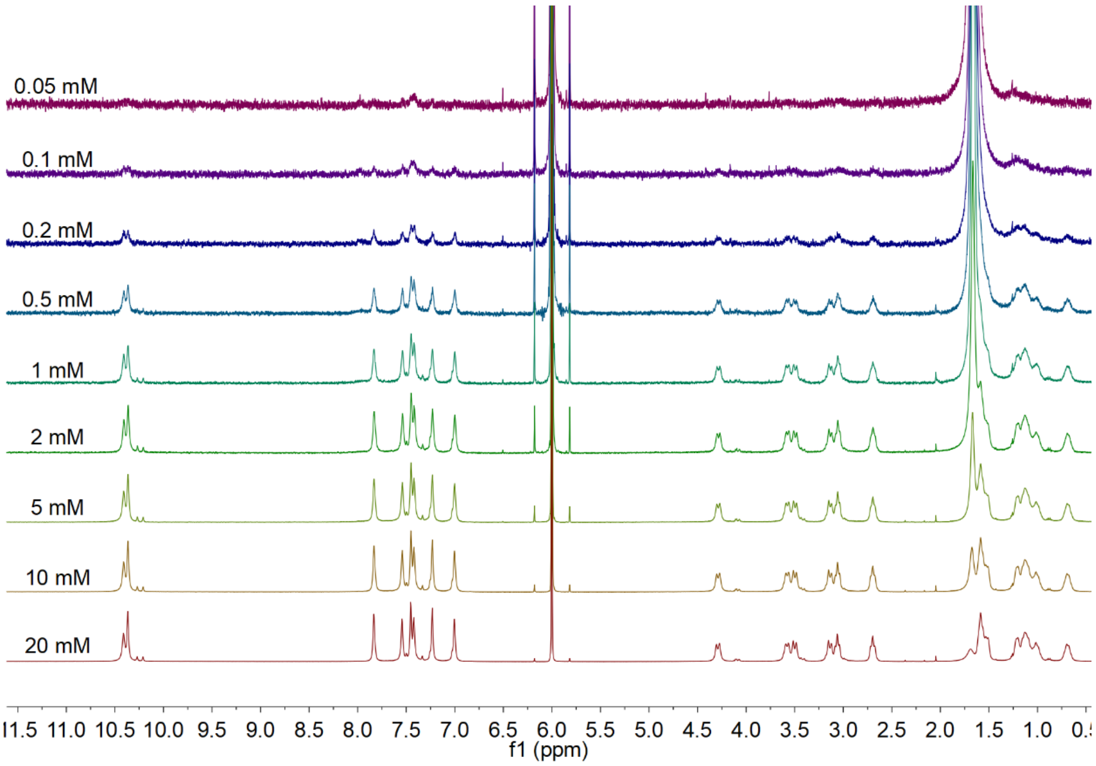

Supplementary Fig. 23 Concentration-variable ¹H NMR (298 K, 500 MHz, $(CDCl_2)_2$)) of $\mathbf{M}_2$. The numbers on the left indicated the concentrations of the macrocycle **M** used to prepare the solutions.

Temperature-variable ¹H NMR studies. Temperature-variable ¹H NMR spectra of **M**$_2$ and Xe ⊂ **M**$_2$ were recorded at every 5 deg interval (Supplementary Figs. 24 and 25). Upon temperature increasing, the two sets of signals started to broaden and reached coalescence at the end. Comparing to the free **M**$_2$ sample, the Xe ⊂ **M**$_2$ complex showed a slower broadening process and a higher coalescence temperature. Upon temperature increasing, the chemical shifts of the N*H* protons didn't change to a large extent, suggesting the dimeric form of the macrocycle was maintained (large upfield shifts of the N*H* proton signals would be observed if the dimer had disassembled). Also in the spectra of Xe ⊂ **M**$_2$ sample, upon temperature increasing, a set of peaks corresponding to the free **M**$_2$ species gradually emerged (due to the escape of xenon from the capsule), and coexisted with the signals of Xe ⊂ **M**$_2$ species, but no signals for the free macrocycle species were observed. These results suggested the broadening and coalescence of the two sets of signals should reflect an acceleration of the two-site position-exchange process, rather than the decomposition of the dimer to produce significant amounts of free macrocycle.

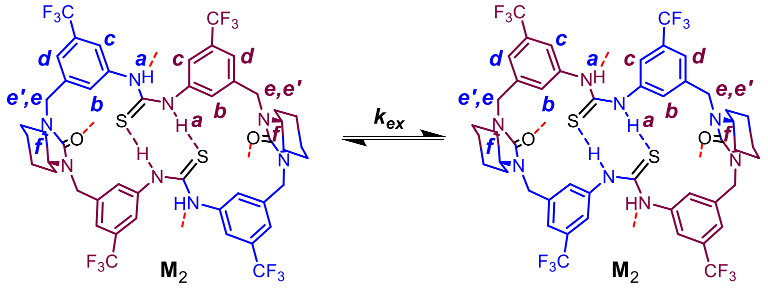

The activation energy ΔG‡ for the exchange between the two equally populated sites can be determined according to the below equation, whereas T_c is the coalescence temperature and Δv corresponds to the difference of the chemical shifts in hertz between the two signals in the absence of exchange.[3] Here the difference of the chemical shifts at 233 K was used as Δv for calculation as the value reached almost constant at these low temperatures (Supplementary Table 6).

$$\Delta G^{\ddagger} = RT_c\left[22.96 + \ln\left(\frac{T_c}{\Delta v}\right)\right] \qquad \text{Supplementary Equation (2)}$$

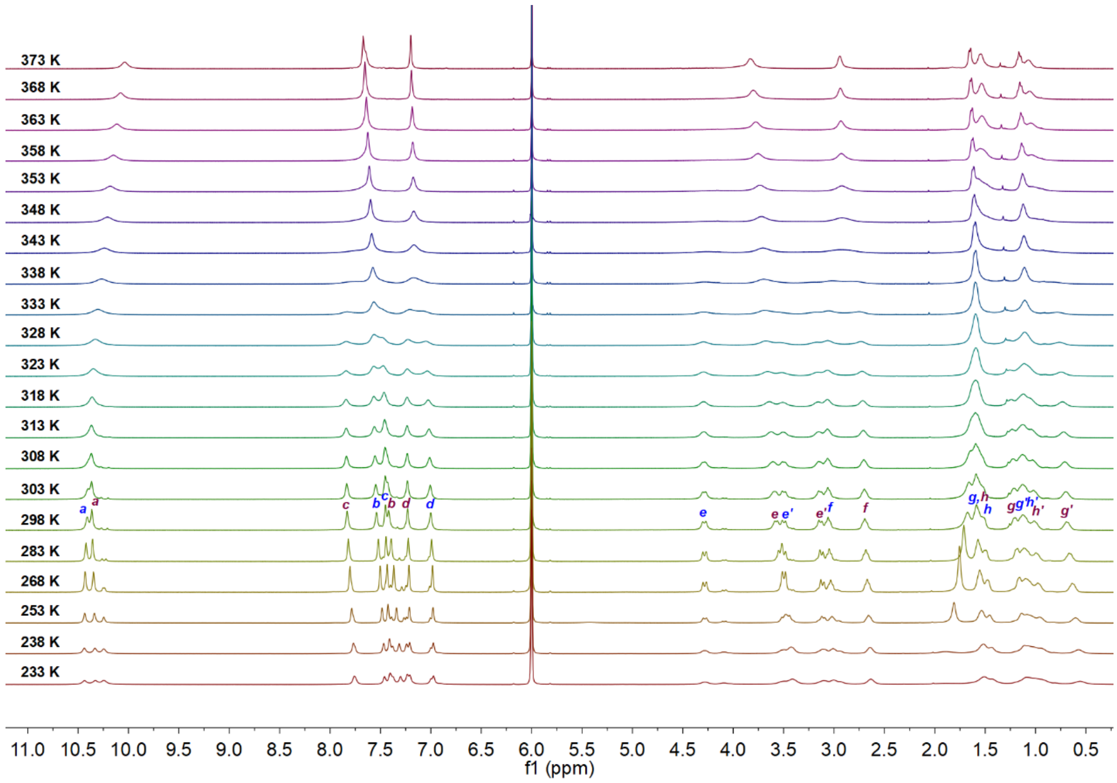

Supplementary Fig. 24 Temperature-variable ^{1}H NMR (500 MHz, $(CDCl_2)_2$) of $\mathbf{M}_2$ ($[\mathbf{M}]_{initial}$ = 10 mM).

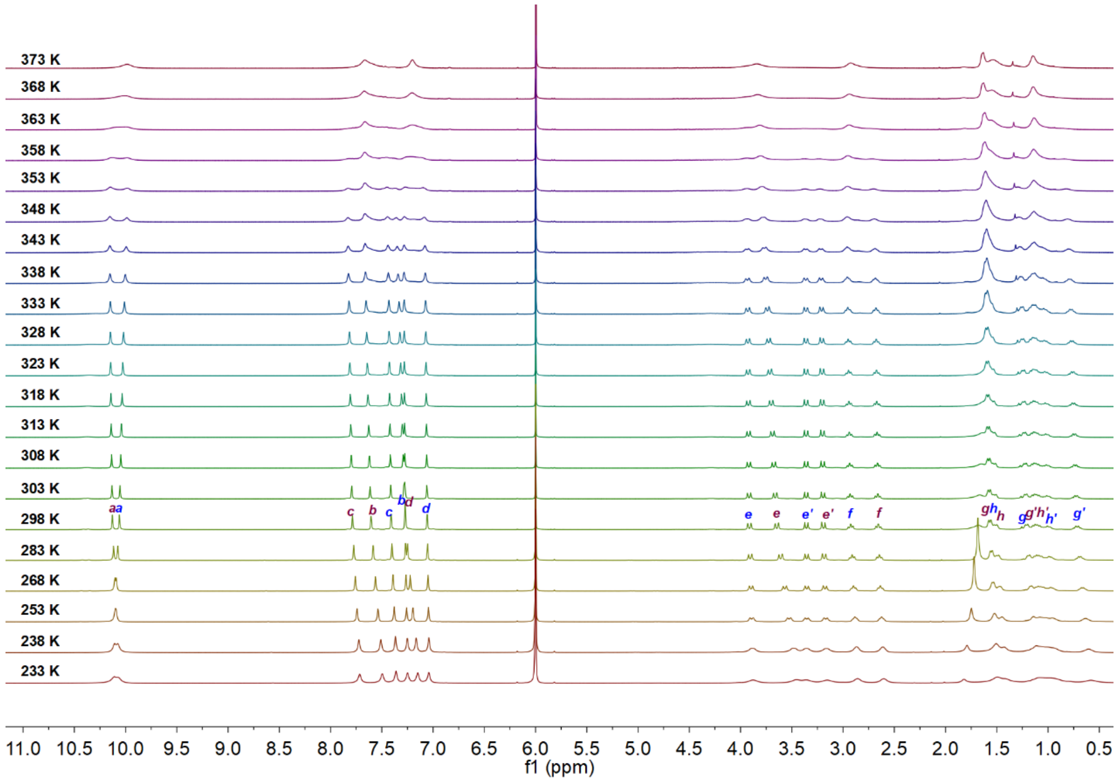

Supplementary Fig. 25 Temperature-variable ^{1}H NMR (500 MHz, $(CDCl_2)_2$) of Xe $\subset$ $\mathbf{M}_2$ (obtained through bubbling of xenon to a solution of $\mathbf{M}_2$) ($[\mathbf{M}]_{initial}$ = 10 mM). “*” denotes the emerging peaks of free $\mathbf{M}_2$.

Supplementary Table 6. Determination of the activation energy $\Delta G^{\ddagger}$ for the two-site position-exchange.

	Protons	$\delta_{T = 233\ K}$ (ppm)	$\Delta\nu$ (Hz)	T_c (K)	$\Delta G^{\ddagger}$ (kcal mol^{-1})	$\Delta G^{\ddagger}_{avg}$ (kcal mol^{-1})
	b and ***b***	7.460, 7.301	79.5	338	16.4	
$\mathbf{M}_2$	***c*** and ***c***	7.403, 7.756	176.5	353	16.6	16.4 ± 0.2
	d and ***d***	6.973, 7.206	116.5	338	16.1	
	b and ***b***	7.150, 7.495	172.5	368	17.3	
Xe ⊂ $\mathbf{M}_2$	***c*** and ***c***	7.362, 7.716	177.0	368	17.3	17.4 ± 0.1
	d and ***d***	7.042, 7.249	103.5	363	17.5	

ITC studies. In order to obtain the self-association dimerization constant ($K_{dimerization}$) of the macrocycle **M**, we carried out ITC dilution titrations (Supplementary Fig. 26). All measurements were performed in a VP-ITC microcalorimetric system (Malvern) with a stainless steel sample cell of 1.4319 mL at 25.00 °C. The number of injections was 28, and the volume of each injection was 10 μL with 20 s of duration and 150 s of spacing between the injections. The pure 1,1,2,2-tetrachloroethane solvent was loaded into the sample cell of the microcalorimeter. The prior prepared dimer solution ($[\mathbf{M}]_{initial}$ = 1 mM) was loaded into an injection syringe (0.280 mL), and was gradually titrated into the pure tetrachloroethane solvent. The collision heat was measured by titrating pure tetrachloroethane solvent into the pure tetrachloroethane solvent following similar operation. The collision heat curve was subtracted from each dilution titration curve. Data were analyzed with the NanoAnalyze software (TA Instruments) and fitted with dimer dissociation model. The dissociation constant (K_d) and enthalpy change ($\Delta H°$) were derived when the best fitting was obtained. Two runs of experiments were carried out and averaged values of these parameters were adopted.

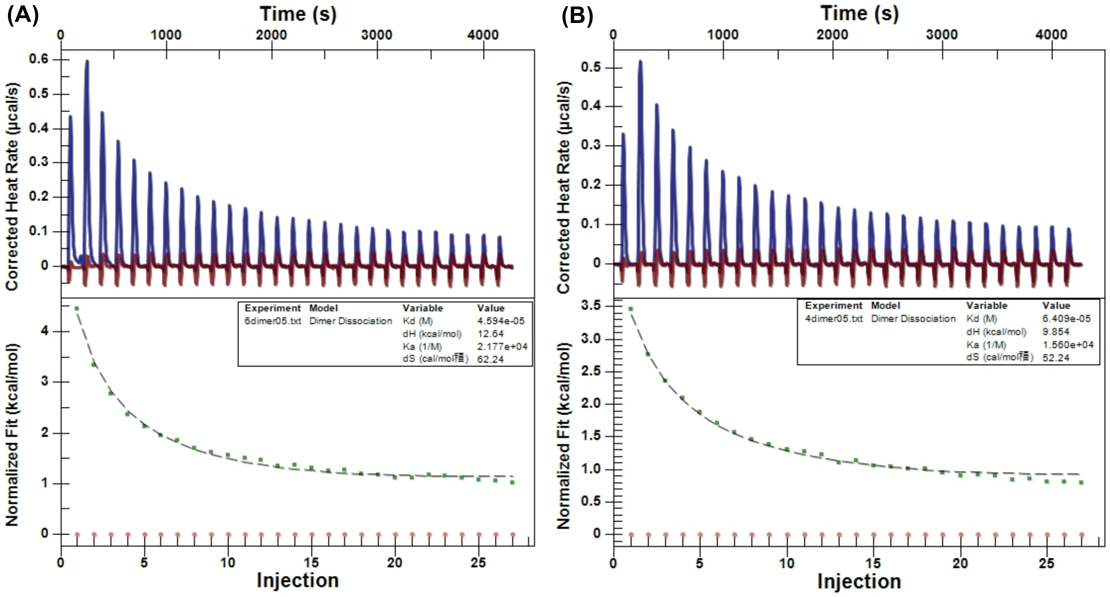

Supplementary Fig. 26 ITC titration curves of the dilution of **M**$_2$ ($(CHCl_2)_2$, 298 K). Raw data for sequential injections of the dimer solution ([**M**]$_{initial}$ = 1 mM, blue) or pure 1,1,2,2-tetrachloroethane solvent (purple) into pure 1,1,2,2-tetrachloroethane solvent (upper panel). Net heat effect of dissociation of the dimer by subtracting the heat of collision (dotted line) and the fitted curve (solid line) (lower panel). Two runs of experiments were carried out (A and B). The calculated average dissociation constant $K_d = (5.5 \pm 0.9)\times10^{-5}$ M, dimerization constant $K_{dimerization} = (1.9 \pm 0.3)\times10^{4}$ M^{-1}, $\Delta G° = -5.8 \pm 0.1$ kcal mol^{-1}.

HR CSI-MS study. The high-resolution CSI-MS study was carried out on a solution of **M** in acetonitrile at room temperature. In the spectrum both the peaks corresponding to the monomeric species [**M**-H]$^-$ and the dimeric species [**M**$_2$-H]$^-$ were observed, whereas the latter is dominant (Supplementary Fig. 27).

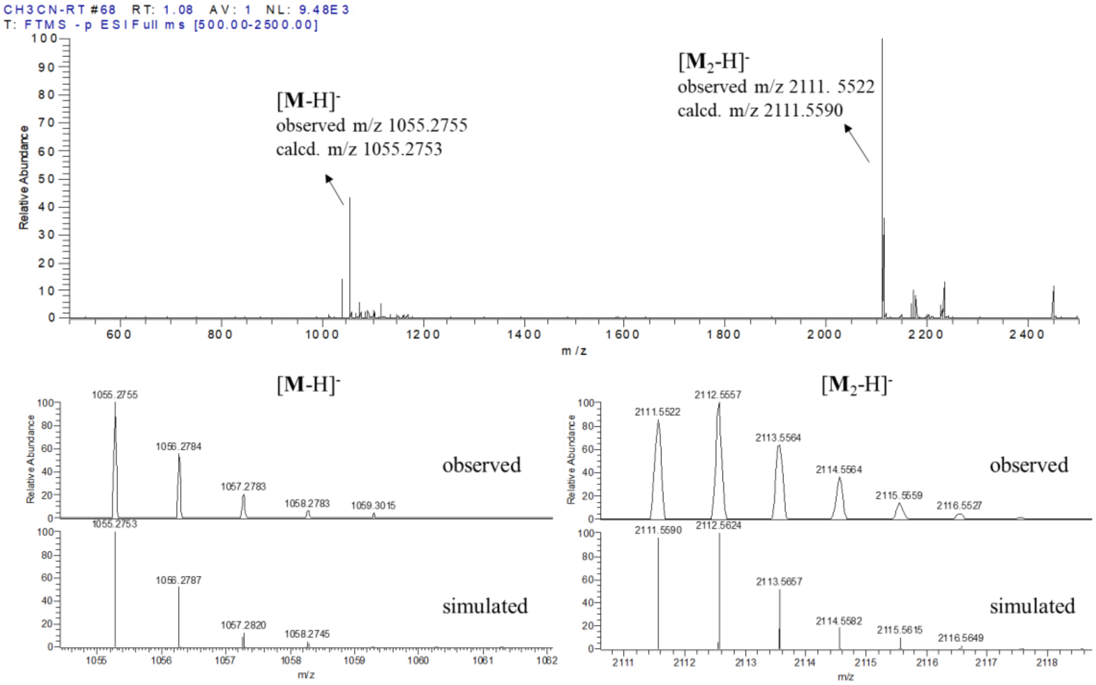

Supplementary Fig. 27 HR CSI-MS (negative mode, 298 K) of **M**$_2$. Top, full spectrum; bottom, the observed and simulated spectra for each individual species.

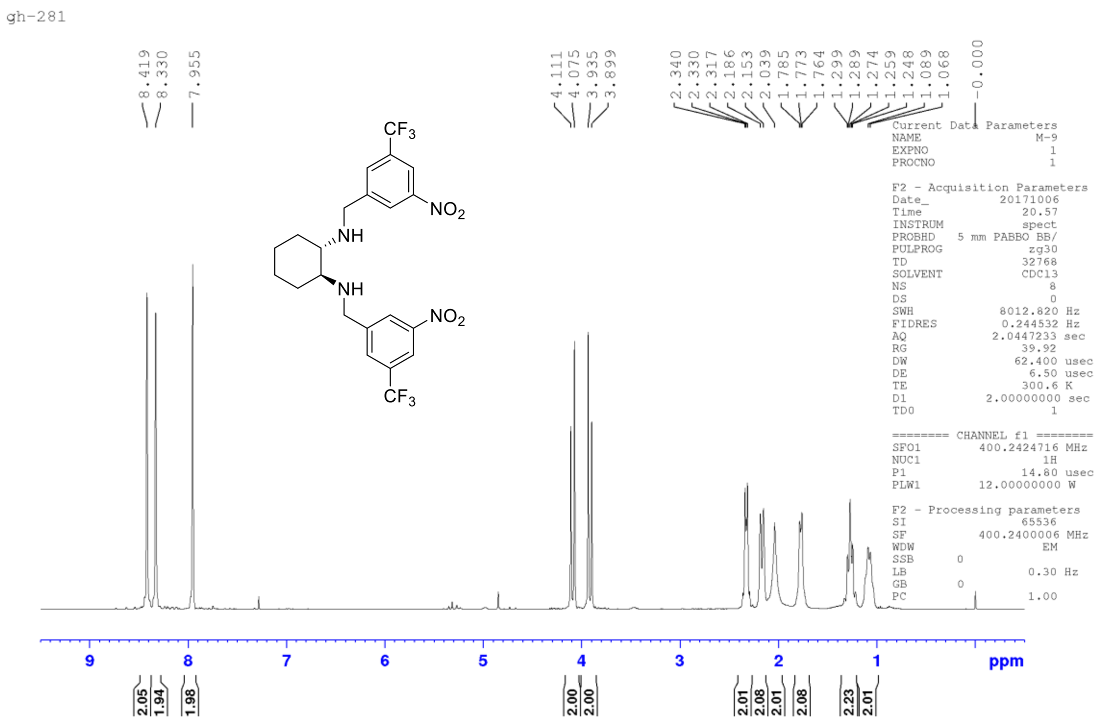

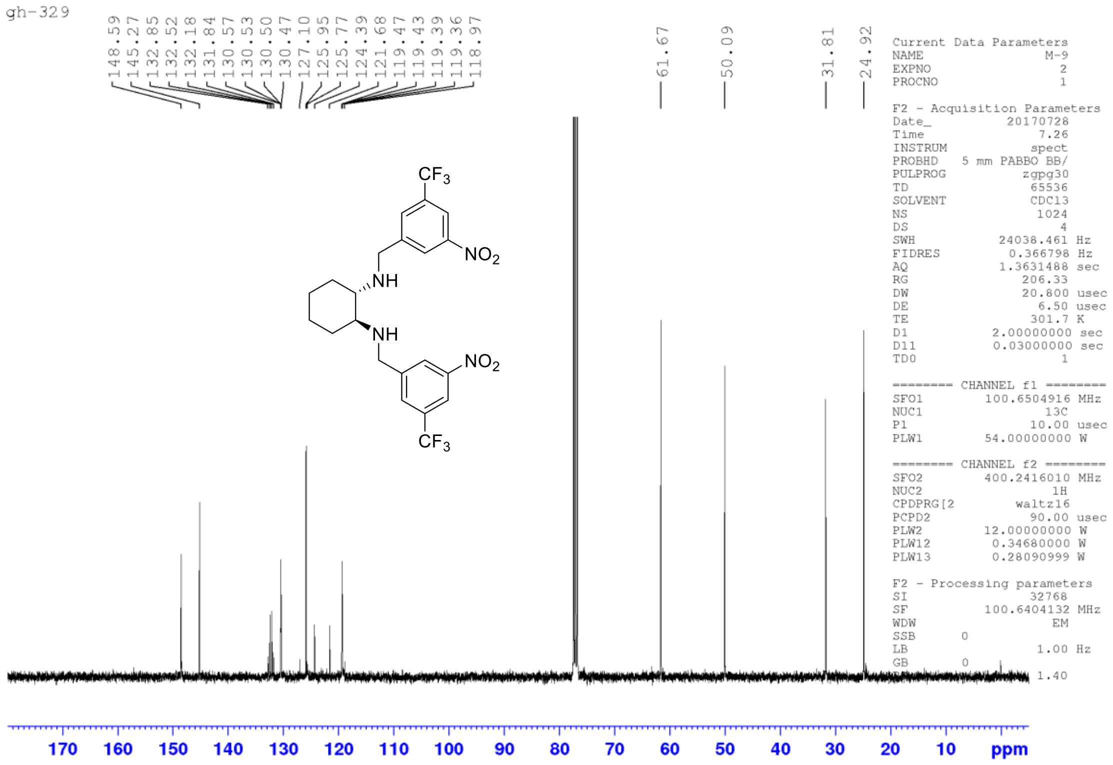

Supplementary Fig. 28 ^{1}H and ^{13}C NMR of **5** in $CDCl_3$.

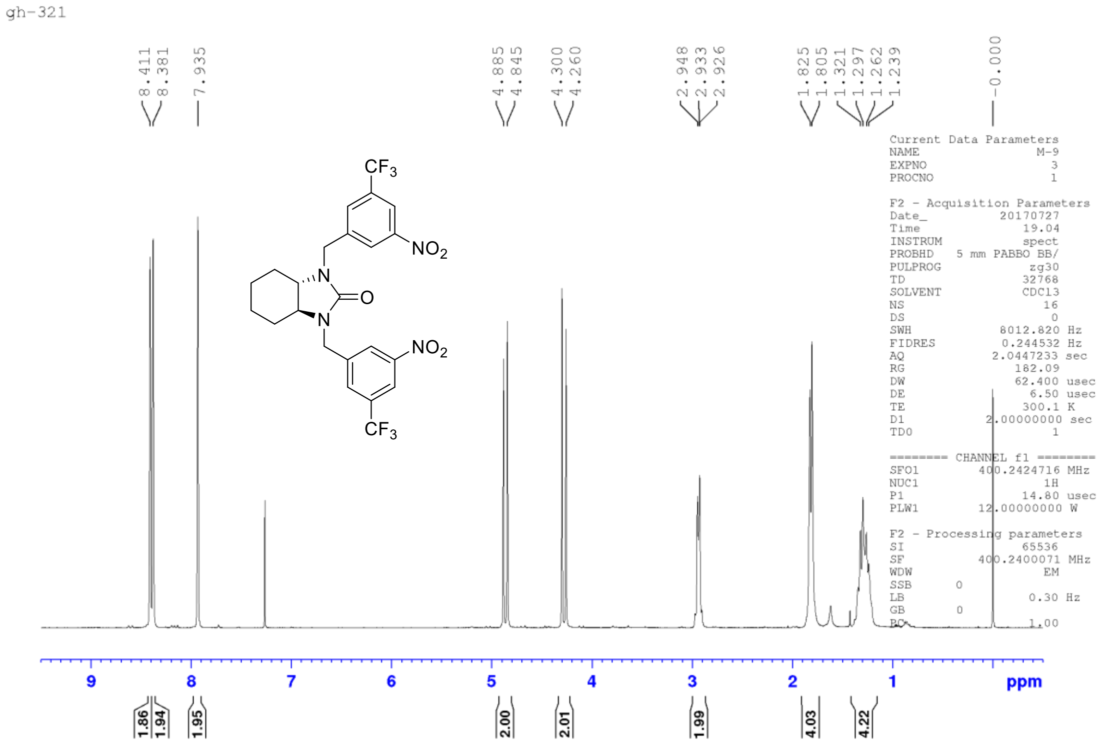

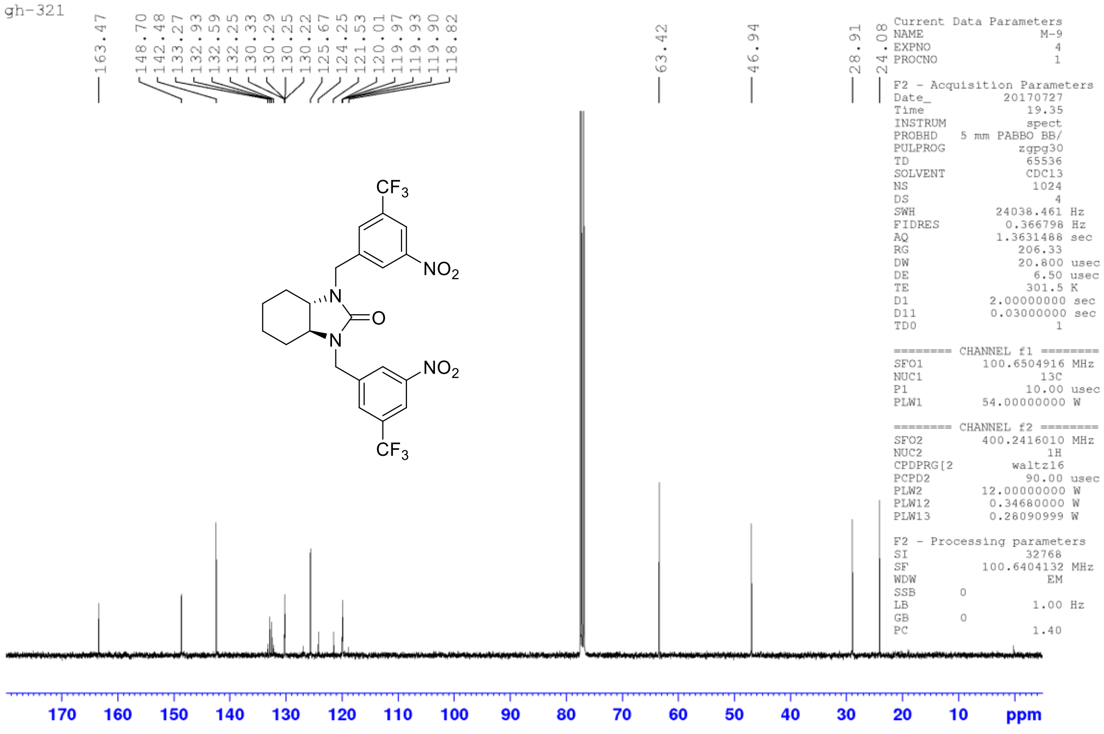

Supplementary Fig. 29 ^{1}H and ^{13}C NMR of **6** in $CDCl_3$.

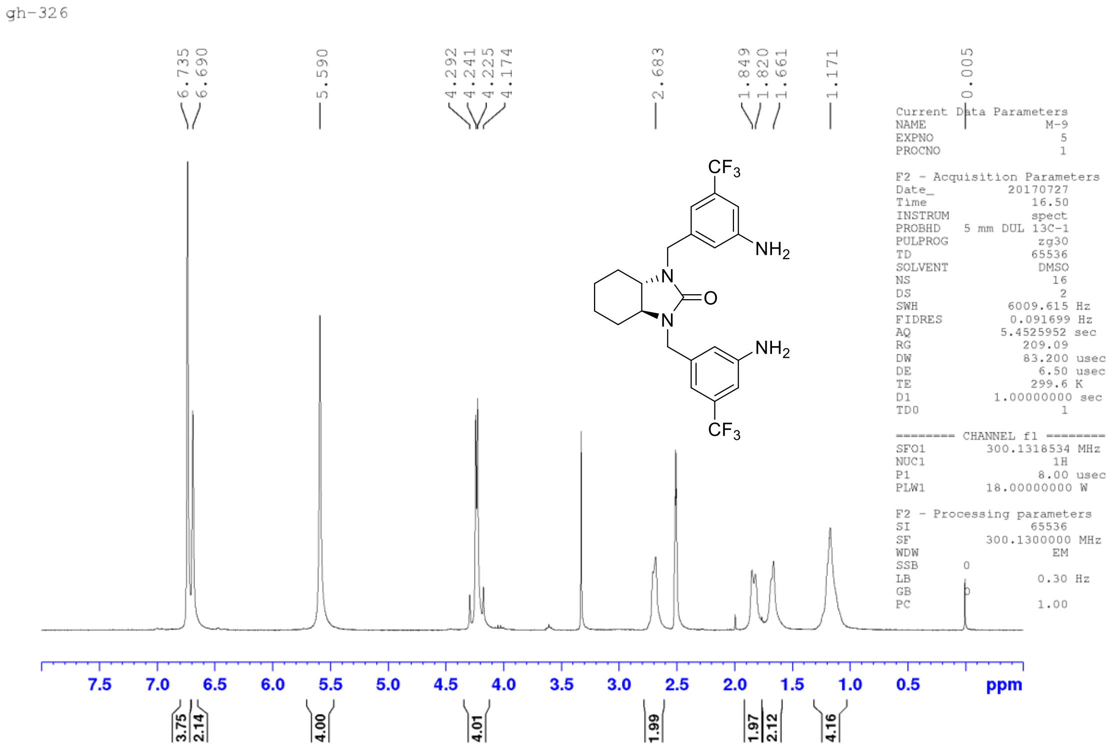

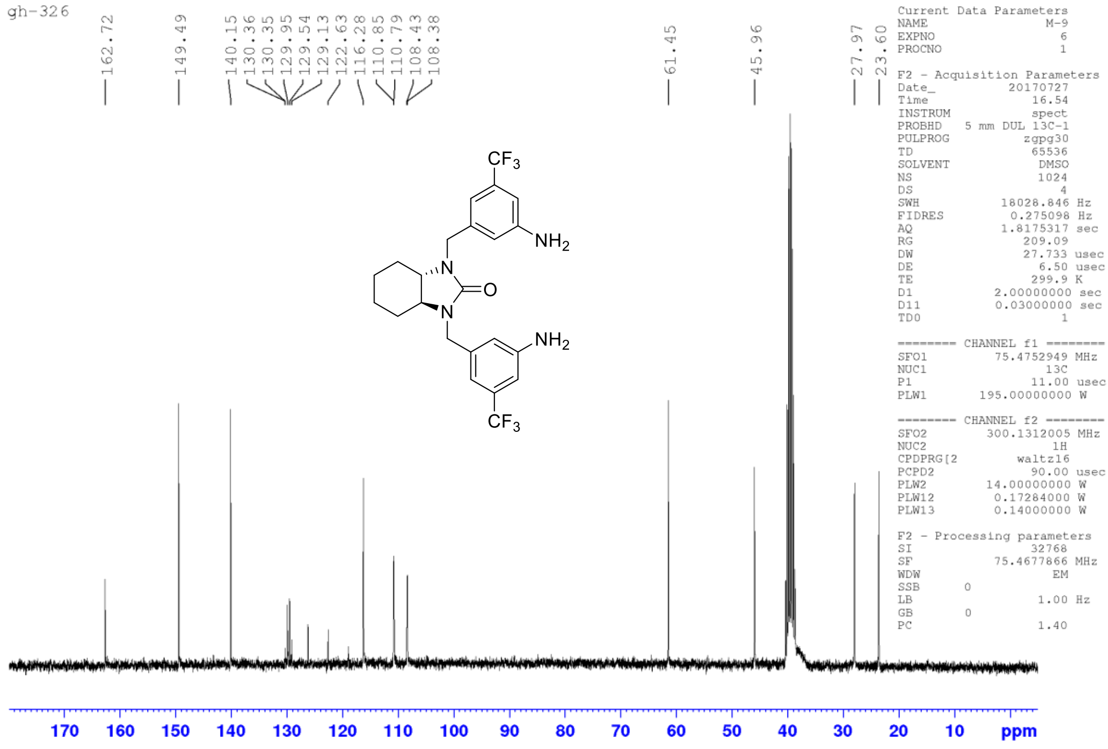

Supplementary Fig. 30 ^{1}H and ^{13}C NMR of **1** in DMSO-d_6.

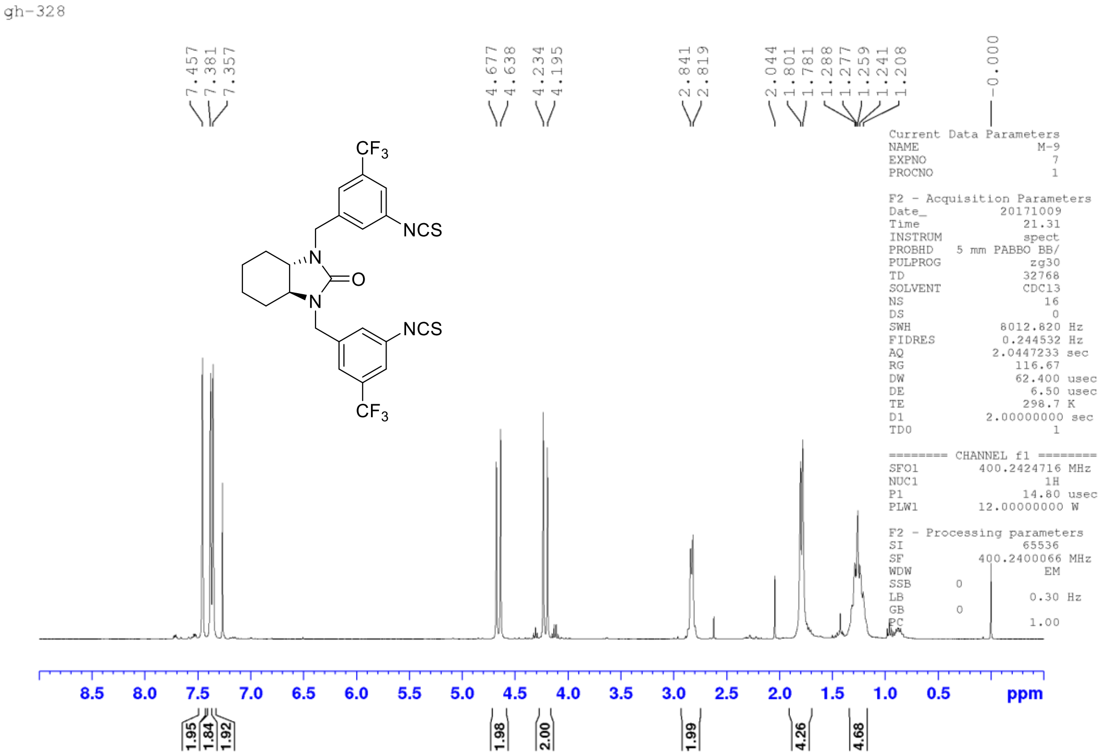

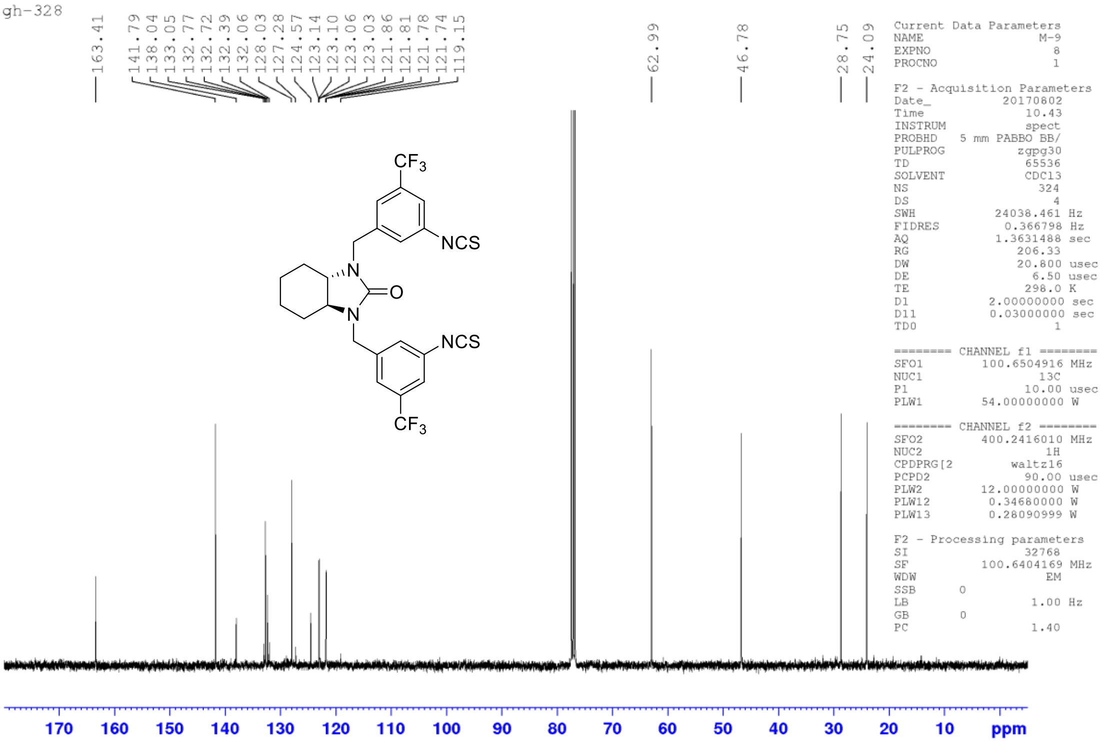

Supplementary Fig. 31 ^{1}H and ^{13}C NMR of **2** in $CDCl_3$.

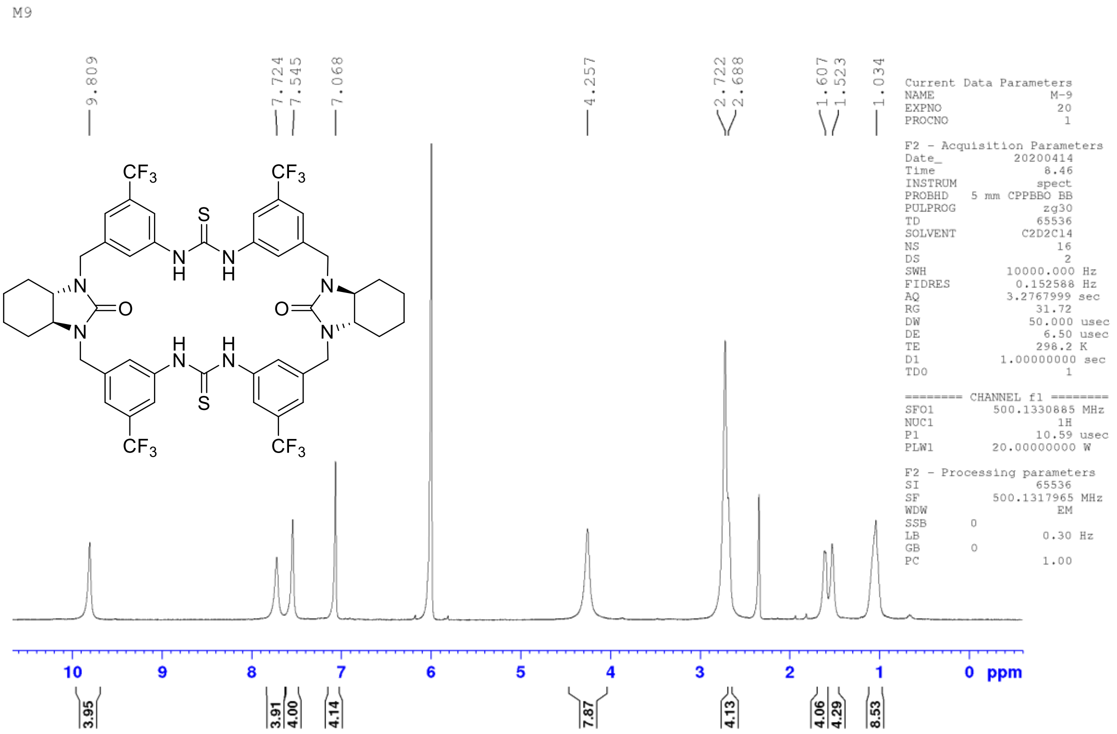

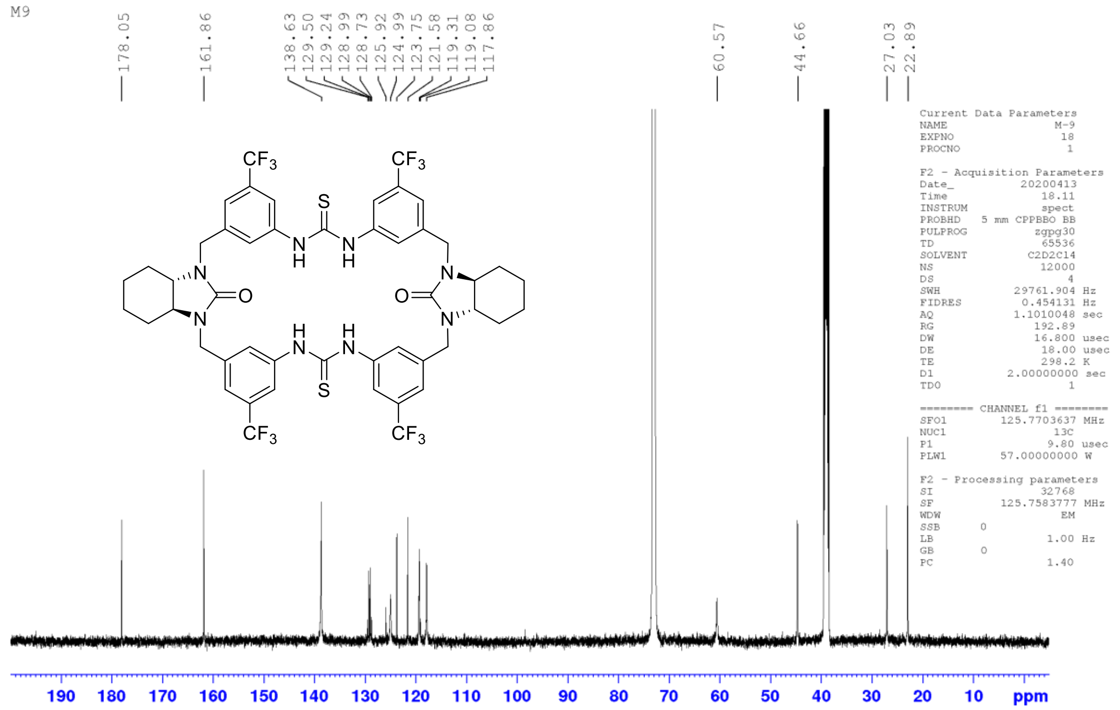

Supplementary Fig. 32 ^{1}H and ^{13}C NMR of **M** in $(CDCl_2)_2$/DMSO-d_6 (5:1).

Supplementary Note 1

In order to roughly assess the sequestration ability of the crystal capsule toward xenon gas, we have built the below thermodynamics cycle and obtained the dissociation pressure for xenon bound within the crystal capsule ($P_{\mathrm{dissociation}}$ = 0.1 atm).

Xe ⊂ **M2** (s) → Xe (g) + **M2** (s) $\Delta G_1 = \Delta G_2 + \Delta G_3 + \Delta G_4 - \Delta G_5$

Supplementary Equation (3)

Xe ⊂ **M2** (solv) → Xe (solv) + **M2** (solv) ΔG_2 = 2.7 kcal/mol ($K_d = 1/K_a = 0.01$)

Supplementary Equation (4)

Xe (solv) → Xe (g) ΔG_3 = -1.4 kcal/mol (K_{evap} = 10)   Supplementary Equation (5)

M2 (solv) → **M2** (s) ΔG_4 Supplementary Equation (6)

Xe ⊂ **M2** (solv) → Xe ⊂ **M2** (s) ΔG_5 Supplementary Equation (7)

Assuming $\Delta G_4 = \Delta G_5$ (as upon solvation, the interior sealed guest would not interact with the solvent, so the solvation processes for the empty and occupied capsule could be assumed to have the same energetics), then $\Delta G_1 = \Delta G_2 + \Delta G_3$ = 1.3 kcal/mol, K_{seq} = 0.1, thus $P_{\mathrm{dissociation}}$ = 0.1 atm.

Based on the extremely low concentration of xenon in atmosphere (0.087 ppmv), uptake of xenon by the capsule directly from the atmosphere is unfavorable based on above estimation of the xenon association pressure. However, in our case, as the xenon is completely sealed within the enclosed cavity of the capsule and cannot be easily escaped, thus the Supplementary Equation (3) could not be considered as a real equilibrium. Therefore the capability for uptake of xenon as a crystalline solid should be associated to the situation in solution. During the crystal cultivation, xenon-saturated capsule solution was used, and in this solution the ratio for xenon-occupied to empty capsule is about 10.5:1 (from NMR integration), which is actually very close to the

xenon occupancy ratio in the obtained crystal. As the current xenon binding affinity in solution is about 100 M^{-1}, the sequestration of atmospheric xenon into solution (and then capture by crystallization) should be also infeasible. In this sense, to our knowledge there are currently no any kind of host materials (as either solid or solution) competent for sequestration of xenon from atmosphere directly. What we suppose is that development of xenon host materials (like our system) could have the potential for the separation of xenon from the already-enriched sample (e.g. a mixture of xenon and krypton) after using the traditional cryogenic methods.[4-5]

Supplementary References

1. Bartik, K.; Luhmer, M.; Dutasta, J.-P.; Collet, A.; Reisse, J. ^{129}Xe and ^{1}H NMR Study of the Reversible Trapping of Xenon by Cryptophane-A in Organic Solution. *J. Am. Chem. Soc.* **1998**, *120*, 784-791.
2. Segebarth, N.; Aïtjeddig, L.; Locci, E.; Bartik, K.; Luhmer, M. Novel Method for the Measurement of Xenon Gas Solubility Using ^{129}Xe NMR Spectroscopy. *J. Phys. Chem. A* **2006**, *110*, 10770-10776.
3. Günther, H. *NMR Spectroscopy: Basic Principles, Concepts and Applications in Chemistry, 3rd Edition*. Wiley-VCH: Weinheim, 2013, pp.501-509.
4. Kerry, F. G. *Industrial Gas Handbook: Gas Separation and Purification*, CRC: Boca Raton, 2007.
5. Ying, R. T. *Gas Separation by Adsorption Processes*, Butterworth-Heinemann: Oxford, 2013.
